# Complexity trade-offs and equi-complexity in natural languages: a meta-analysis

**DOI:** 10.1515/lingvan-2021-0054

**Published:** 2022-10-14

**Authors:** Christian Bentz, Ximena Gutierrez-Vasques, Olga Sozinova, Tanja Samardžić

**Affiliations:** Department of General Linguistics, University of Tübingen, Tübingen, Germany; URPP Language and Space, University of Zürich, Zürich, Switzerland

**Keywords:** complexity measures, complexity trade-offs, equi-complexity, language complexity

## Abstract

In linguistics, there is little consensus on how to define, measure, and compare complexity across languages. We propose to take the diversity of viewpoints as a given, and to capture the complexity of a language by a vector of measurements, rather than a single value. We then assess the statistical support for two controversial hypotheses: the trade-off hypothesis and the equi-complexity hypothesis. We furnish meta-analyses of 28 complexity metrics applied to texts written in overall 80 typologically diverse languages. The trade-off hypothesis is partially supported, in the sense that around one third of the significant correlations between measures are negative. The equi-complexity hypothesis, on the other hand, is largely confirmed. While we find evidence for complexity differences in the domains of morphology and syntax, the overall complexity vectors of languages turn out virtually indistinguishable.

## 1 Introduction

Some of the fiercest linguistic debates revolve around the complexity of languages: how to define it, how to measure it, and how to compare it between languages. Several volumes and special issues have been dedicated to these questions in the last decades (Baechler and Seiler 2016; Baerman et al. 2015; Miestamo et al. 2008; Newmeyer and Preston 2014; Sampson et al. 2009).

Despite the concerted efforts of researchers to come to grips with these questions, it is safe to say that little consensus has been reached. In particular, there have been no generally accepted definitions of complexity emerging, either for particular domains, e.g., morphology and syntax, or for the “wild goose” (Deutscher 2009) of overall complexity. Given this state of affairs, our proposal is to drop the idea of developing the single “right” definition of complexity, and instead to acknowledge that linguists are diverse in their views of complexity. In this spirit, we use here a variety of complexity metrics to reflect the diversity of definitions, and to shed some light on two fundamental questions:

1. How much evidence do we find for complexity *trade-offs*?
2. How much support is there for the so-called *equi-complexity* hypothesis?

## 2 Trade-offs versus equi-complexity: theoretical background

In the context of language complexity research, one of the most frequently cited statements goes back to Charles Hockett:

Objective measurement is difficult, but impressionistically it would seem that the total grammatical complexity of any language, counting both morphology and syntax, is about the same as that of any other. (Hockett 1958, 180)

This has become known as the *equi-complexity hypothesis*. In Hockett’s original publication another statement follows right after:

Thus one scale for the comparison of the grammatical systems of different languages is that of average degree of morphological complexity – carrying with it an inverse implication as to degree of syntactical complexity. (Hockett 1958, 181)

This is termed the *trade-off hypothesis*. We will here not attempt to give an exhaustive review of all the literature relating to these two hypotheses. Some recent overview articles include Miestamo (2017) and Kortmann and Schröter (2020). Suffice it to say that, over the years, these two statements have become intertwined and are viewed by many researchers as essentially two sides of the same coin: trade-offs logically entail equi-complexity.

However, if formulated mathematically, this entailment turns out to be false. In Appendices 1 and 2,1All appendices can be found at https://github.com/christianbentz/ComplexityMetaAnalyses. we prove this by elaborating on a point made already in Fenk-Oczlon and Fenk (2011, 2014 and Sinnemäki (2014), namely, that negative correlations between complexity values in different domains do not strictly entail equal overall complexity for languages. In other words, finding negative correlations (even perfect ones) between morphological and syntactic complexity measurements *does not* strictly entail equality of mean complexity by language. As a practical consequence, in statistical accounts, we should test for trade-offs and equi-complexity separately.

## 3 Data used for meta-analyses

### 3.1 Text and language samples

We here harness the results of the first Interactive Workshop on Measuring Language Complexity (IWMLC) held in Freiburg in 2019.2
http://www.christianbentz.de/MLC2019_index.html. Participants of the workshop chose a “track”, i.e. a corpus to work with. Participants of Track A worked with 49 translations of the Parallel Bible Corpus (Mayer and Cysouw 2014). This sample of texts represents 49 typologically diverse languages from different areas and families. Participants of Track B, on the other hand, worked with 63 Universal Dependency (UD) treebanks (Nivre et al. 2017), which correspond to 44 different languages. The language sample of this track is less balanced, including many Indo-European languages (i.e. 31 or 70%). However, as an upside, the richer annotations of the UD allow more detailed analyses. World maps with an overview of the language samples are given in Figure 1.3The R code to produce this figure is given in Appendix 3. Overall, these two samples together amount to 80 languages belonging to 34 language families.

**Figure 1:**
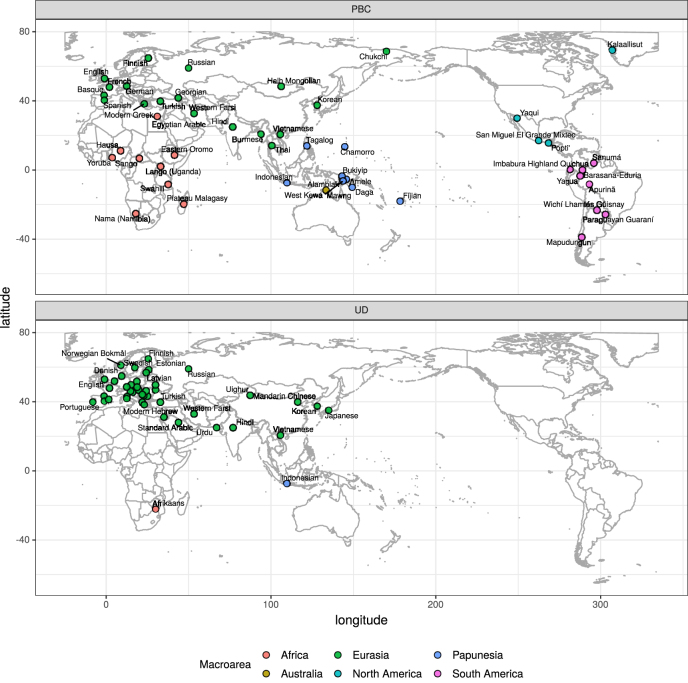
World map with language samples of Track A (Parallel Bible Corpus, top panel) and Track B (Universal Dependencies, bottom panel). Macroareas from Glottolog (Hammarström et al. 2020) are indicated with colors.

### 3.2 Complexity measures

Given the text samples in Tracks A and B, overall seven teams of researchers engaged in the shared task of estimating the languages’ complexities – defining and applying measures of their choice. However, to ensure reproducibility, two important requirements had to be met: a) to use one of two pre-defined corpora, b) to publish the implemented code openly. An overview of the proposed complexity measures is given in Table 1. The results, code, and more detailed meta-information can be found on github.4
https://github.com/IWMLC/language-complexity-metrics. Based on what the respective authors write about their measure(s), these are assigned to two domains, i.e. morphology or syntax.

**Table 1:** Complexity measures implemented for the shared task of the IWMLC workshop. The column “inclusion” indicates which measures were used for the current analyses (✓), and which were discarded (×).

Author(s)	Measure	Inclusion	Short description	Domain	Source	Track
Gutierrez-Vasques & Mijangos (GM)	GM_H1gram	×	Sub-word entropy rate at the character level	Morphology	PBC	A
	GM_H3gram	×	Sub-word entropy rate at the tri-character level	Morphology	PBC	A
	GM_TTR	×	Type-token-ratio	Morphology	PBC	A
	GM_TTR.H1	×	Combination of GM_TTR and GM_H1gram	Morphology	PBC	A
	GM_TTR.H3	×	Combination of GM_TTR and GM_H3gram	Morphology	PBC	A
	GM_TTR.H1.H3	×	Combination of GM_TTR, GM_H1gram, and GM_H3gram	Morphology	PBC	A
	GM_H1gram_fullyparallelised	✓	Same as GM_H1gram but with fully parallel bible verses	Morphology	PBC	A
	GM_H3gram_fullyparallelised	✓	Same as GM_H3gram but with fully parallel bible verses	Morphology	PBC	A
	GM_TTR_fullyparallelised	✓	Same as GM_TTR but with fully parallel bible verses	Morphology	PBC	A
	GM_TTR.H1_fullyparallelised	×	Same as GM_TTR.H1 but with fully parallel bible verses	Morphology	PBC	A
	GM_TTR.H3_fullyparallelised	×	Same as GM_TTR.H3 but with fully parallel bible verses	Morphology	PBC	A
	GM_TTR.H1.H3_fullyparallelised	×	Same as GM_TTR.H1.H3 but with fully parallel bible verses	Morphology	PBC	A
Oh (OH)	OH_MC	✓	Morphological complexity based on WALS features	Morphology	WALS	A
	OH_SID	✓	Syllable information density	Morphology	PBC	A
	OH_WD	✓	Word information density	Morphology	PBC	A
Brunato & Venturi (BV)	BV_n_tokens	✓	Sentence length	Syntax	UD	B
	BV_char_per_tok	✓	Word length	Morphology	UD	B
	BV_verbal_head_per_sent	✓	Distribution of verbal head	Syntax	UD	B
	BV_verbal_root_perc	✓	Distribution of verbal roots	Syntax	UD	B
	BV_avg_token_per_clause	×	Clause length	Syntax	UD	B
	BV_avg_links_len	✓	Length of dependency links	Syntax	UD	B
	BV_avg_max_depth	×	Depth of parse tree	Syntax	UD	B
	BV_avg_verb_edges	✓	Verb arity	Syntax	UD	B
	BV_avg_subordinate_chain_len	✓	Average depth of chains of embedded subordinate clauses	Syntax	UD	B
	BV_avg_subordinate_pre	✓	Distribution of subordinate clauses (preceding main clause)	Syntax	UD	B
	BV_avg_subordinate_post	✓	Distribution of subordinate clauses (following main clause)	Syntax	UD	B
Çöltekin & Rama (CR)	CR_inflection_accuracy	✓	Inflection_accuracy	Morphology	UD	B
	CR_ttr	✓	Type-token-ratio	Morphology	UD	B
	CR_msp	✓	Mean size of paradigm	Morphology	UD	B
	CR_mfe	✓	Morphological feature entropy	Morphology	UD	B
	CR_cfe_form_feat	✓	Conditional entropy of form given feature	Morphology	UD	B
	CR_cfe_feat_form	✓	Conditional entropy of feature given form	Morphology	UD	B
Semenuks (S)	S_idMean	✓	Average information density	Syntax	UD	B
	S_SidSD	✓	Standard deviation of the information content	Syntax	UD	B
Sinnemäki & Haakana (SI)	SI_dm	✓	Percentage of possessive noun phrases with dependent marking	Morphology	UD	B
	SI_hm	✓	Percentage of possessive noun phrases with head marking	Morphology	UD	B
	SI_dep_dl	✓	Dependency length in possessive NP with marking on dependent	Syntax	UD	B
	SI_double_dl	×	Dependency length in possessive NP with marking on dependent and head	Syntax	UD	B
	SI_head_dl	×	Dependency length in possessive NP with marking on head	Syntax	UD	B
	SI_zero_dl	×	Dependency length in possessive NP with zero marking	Syntax	UD	B
Sozinova, Bentz & Samardžić (SBS)	SBS_INF	✓	Inflectional morphology	Morphology	UD	B
	SBS_DER	✓	Derivational morphology	Morphology	UD	B

Note that for our meta-analyses we exclude 14 of these 42 measures due to them being conceptually redundant or yielding too many NAs. The preprocessing and selection of measures is further described in Appendices 4 and 5. All in all we thus have 28 remaining complexity measures, of which six belong to Track A, and 22 to Track B.

## 4 Methods of meta-analyses

We here follow Deutscher (2009, 247) who proposes to represent the complexity of a language as a vector of complexity values. In this context, we also agree with Miestamo (2008, 30) that *representativity* and *commensurability* are two key issues to keep in mind. Representativity is here achieved by the inclusion of different metrics. Commensurability is ensured by standardization of the data (centering and scaling). The overall complexity of a language x is represented by a vector ${\vec{v}}_{x}$ , where each value is one measurement, such that

(1) $${\vec{v}}_{x}=\left(v_{1},v_{2},\ldots ,v_{n}\right),$$

where n is the overall number of measurements. We can also index values by domain, i.e. m for morphology, and s for syntax. As a simple example take the two vectors in Table 2 with six scaled measurements overall, three of each domain. We use these below for illustration of our methods.

**Table 2:** Made-up example of overall complexity vectors for two languages (A and B).

	$v_{1}^{m}$	$v_{2}^{m}$	$v_{3}^{m}$	$v_{1}^{s}$	$v_{2}^{s}$	$v_{3}^{s}$
${\vec{v}}_{L_{A}}$	−5	−3	−4	4	1	2
${\vec{v}}_{L_{B}}$	−1	0	−2	5	3	6

### 4.1 Testing agreement and trade-offs

We here conceptualize *agreement* between measures as *positive correlations*, and complexity *trade-offs* as *negative correlations*. For instance, if the values in columns $v_{1}^{m}$ and $v_{2}^{m}$ in Table 2 are positively correlated, then the two measures “agree” on how to rate the morphological complexity of language A and language B. On the other hand, if the values in columns $v_{1}^{m}$ and $v_{1}^{s}$ are negatively correlated, this suggests that there is a “trade-off” between these morphological and syntactic complexity measures. We use Spearman rank correlations since, arguably, the ranking of languages in different complexity dimensions is relevant, while the linearity of the relationship between different complexity values is secondary. Also, we adjust for multiple testing by using the Holm-Bonferroni method. More details on how the correlations are calculated and visualized can be found in Appendix 4.

### 4.2 Testing the equi-complexity hypothesis

Recall from Section 2 that correlation tests are not suitable for assessing equi-complexity. Instead, we perform pairwise comparisons of vectors looking for significant *location shifts* in the respective measurements. A shift occurs if the values in one vector tend to be higher than the values in the other vector. To put it the other way around, *equi-complexity* is the lack of location shifts in the complexity vectors of languages. We probe for significant shifts by using Wilcoxon rank sum tests (Wilcoxon 1945). This firstly involves assigning a rank to each scaled measurement from lowest to highest value, as illustrated for our example vectors in Table 3.

**Table 3:** Ranking of example vectors according to complexity values.

Rank	1	2	3	4	5	6	7	8	9	10	11	12
${\vec{v}}_{L_{A}}$	−5	−4	−3				1	2		4		
${\vec{v}}_{L_{B}}$				−2	−1	0			3		5	6

Based on this ranking, the test statistic is defined as the sum of ranks of the vectors (Hollander et al. 2014, 107) such that we have

(2) $$W^{{\vec{v}}_{L_{A}}}=\sum_{i=1}^{k}S_{i}^{{\vec{v}}_{L_{A}}}=31,$$

and

(3) $$W^{{\vec{v}}_{L_{B}}}=\sum_{j=1}^{l}S_{j}^{{\vec{v}}_{L_{B}}}=47.$$

Here, k and l are the number of measurements in the respective vector, and S is the rank of a given complexity value i or j . Following Hollander et al. (2014, 117) the expected value for the sum of ranks under the null hypothesis $\left(H_{0}\right)$ of a random distribution of complexity values (if there are no ties) is

(4) $$E_{0}〚W〛=\frac{k\times \left(l+k+1\right)}{2}=39.$$

The *p*-value then reflects the probability that a given value of W occurs under the null hypothesis that the vectors do not differ in their location. For our example vectors, the *p*-value is 0.24. Hence, while ${\vec{v}}_{L_{B}}$ is shifted to higher values compared to ${\vec{v}}_{L_{A}}$ , this shift is not considered significant. We would conclude that $L_{A}$ and $L_{B}$ are roughly equal in overall complexity.

Note that we use only the languages and measures of Track B for equi-complexity analyses, since here we have both domains – morphology and syntax – represented. For the 44 languages in this track we run pairwise tests, i.e. $\frac{44\times \left(44-1\right)}{2}=946$ tests in total. Again we apply the Holm-Bonferroni correction to adjust *p*-values. Since *p*-values tend to be sensitive to sample sizes, we additionally calculate effect sizes for the pairwise tests. The effect size metric we use here (r) runs from 0 to 1 (Tomczak and Tomczak 2014, 23), with 0.1–0.3 considered a small effect, 0.3–0.5 a medium size effect, and >0.5 a large effect. Further details about the statistical methods can be found in Appendix 5. The *R* code to replicate all of the analyses can be found on github.5
https://github.com/christianbentz/ComplexityMetaAnalyses.


## 5 Results & discussion

### 5.1 Agreement between measures

**Figure 2:**
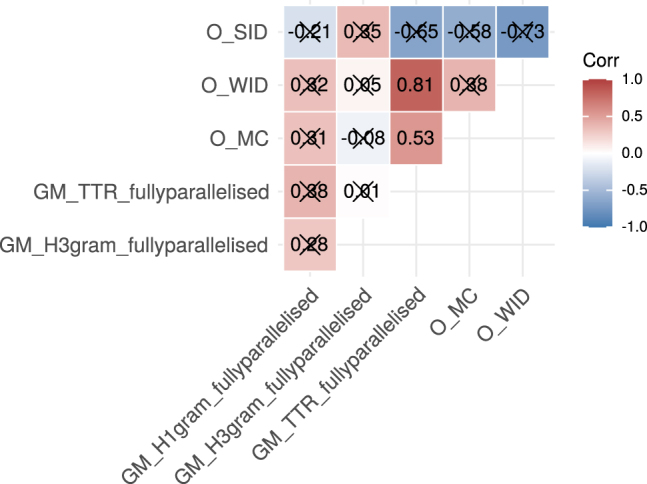
Correlogram with Spearman correlations for complexity measures of Track A (PBC). Correlations which are non-significant after Holm-Bonferroni correction are crossed out.

Firstly, we discuss significant positive Spearman correlations as evidence for agreement between complexity measures. In the correlogram for Track A (Figure 2), we can see that both significant correlations are positive.6When disregarding significance there are 10 positive versus five negative correlations in this track. Note that all of the measures proposed in this track have been assigned to the domain of morphology. There is hence some evidence for agreement between morphological complexity measures.

For example, type-token-ratios (GM_TTR) strongly correlate with word information densities (OH_WID) across the 49 languages of this track. This is further illustrated in panel a of Figure 4. Compare, for instance, the first verse of the New Testament in English (eng) and Kalaallisut (kal):7We glossed this example using the Kalaallisut Grammar by Sadock (2003), and the online dictionary at https://oqaasileriffik.gl/en/.

(1)

**Table:** 

The beginning of the gospel of Jesus Christ, the Son of God;

(2)

**Table:** 

Jiisusi-Kristusimik	Guutip	erneranik	iivangkiiliup	aallaqqaataa.
Jiisusi-Kristusi=mik	Guuti=p	ernerq=mik	iivangkiiliu=p	aallaqqaataa.
Jesus-Christ=INS	God=ERG	son=INS	gospel=ERG	beginning

In the English sentence, we have a TTR of 8/12 = 0.67, while in Kalaallisut this amounts to 6/6 = 1. Note that while English encodes the possessive relationships in this sentence by using the isolating construction “of the”, in Kalaallisut this is encoded by instrumental and ergative inflectional markers. According to the TTR, Kalaallisut is hence more complex than English.

Let us compare the TTR to the so-called *Word Information Density* (WID) as described in Oh (2015, 89). It is computed as the average of the ratio of the number of words per sentence in a baseline language – in this case Thai (tha) – over the number of words in the target language. For our New Testament example this ratio is 13/12 = 1.08 for Thai to English and 13/6 = 2.17 for Thai to Kalaallisut. This tells us that Kalaallisut encodes the same information with fewer word tokens, which hence have higher information density. Thus, it makes sense that the TTR and WID are highly correlated across languages: they both reflect similar dimensions of complexity typically associated with the domain of morphology.

**Figure 3:**
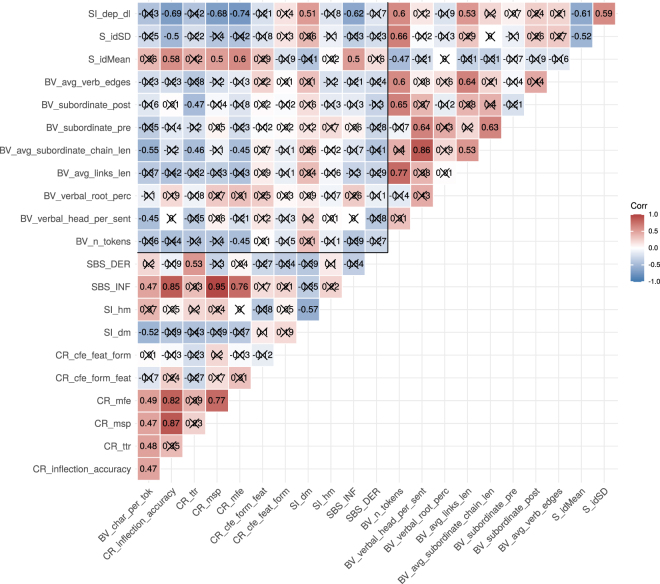
Correlogram with Spearman correlations for complexity measures of Track B (UD). Correlations which are non-significant after Holm-Bonferroni correction are crossed out. The black lines subset the correlogram such that we have correlations between morphological complexity measures in the lower left triangle, those between syntactic complexity measures in the upper right triangle, and those between the domains in the lower right square.

Turing now to Track B, in the correlogram (Figure 3) we find that 29 of overall 43 significant correlations (67%) are positive. So, again there is considerable evidence for agreement between measures. It seems particularly strong between measures assigned to the same domain (i.e. morphology or syntax). Namely, the lower left triangle in Figure 3 gives correlations between morphological complexity measures, and the upper right triangle correlations between syntactic complexity measures. In both corners we see rather positive (red) correlations than negative (blue) correlations (12/14 or 86%, and 12/15 or 80% respectively). To assess whether these correlational trends hold independent of language families, we contrast Indo-European languages with non-Indo-European languages in Appendix 4. The general picture is similar for these family-wise correlograms.

**Figure 4:**
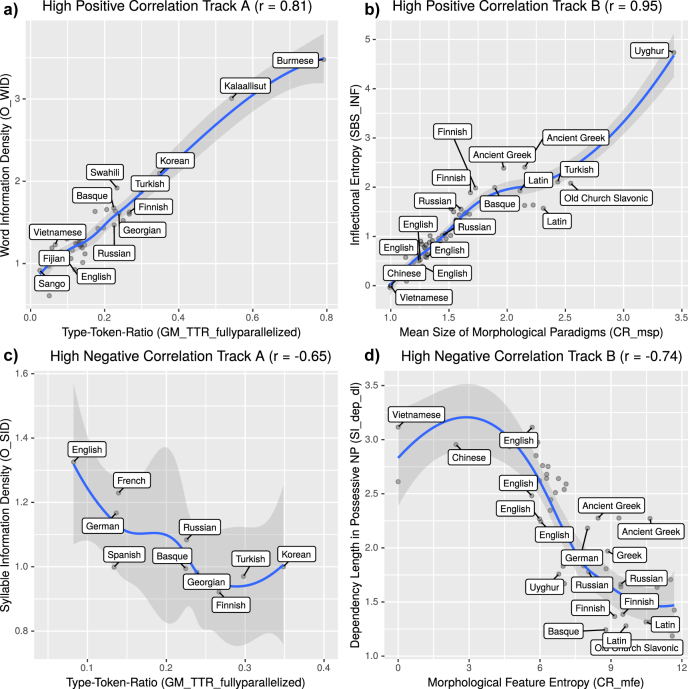
Scatterplots with high positive and negative Spearman correlations in Tracks A and B. Local regression smoothers are given as blue lines with grey confidence intervals.

An example of a very strong (almost perfect) positive Spearman correlation in Track B is found between the *Mean Size of Morphological Paradigms* (CR_msp) and the *Inflectional Entropy* (SBS_INF), as visualized in Figure 4 panel b. In Çöltekin and Rama (2018), the MSP is defined as “the number of word-form types divided by the number of lemma types”. Inflectional entropy, on the other hand, is defined as the entropy of word-form types minus the entropy of lemmas (i.e., when inflections are neutralized). Given that word types and also lemmas are provided for (most) languages in the UD data sets, these measures can be straightforwardly computed.

Take as an example personal pronouns as found in the Old Church Slavonic (chu) texts (Table 4). Different word forms are here all mapped to the same lemma “онъ” (he, ‘on’). For this particular example, we thus get an MSP of 6/1 = 6. If we use the so-called maximum likelihood method for calculating the entropy of word forms and lemmas, then we get an inflectional entropy of 2.58. Compare this to the results of forms and lemmas we get for the corresponding English pronouns (Table 5). Here we have an MSP of 5/3 = 1.67, and an inflectional entropy of exactly 1. Both the MSP and the inflectional entropy would thus rank Old Church Slavonic higher in complexity than English. Again, it makes sense that these measures positively correlate since both hinge upon differences in distributions of word forms versus lemmas.

**Table 4:** Example of Old Church Slavonic pronoun forms and lemmas as found in the UD.

Form	Lemm	Feats	Translation
они	онъ	Case = Nom|Gender = Masc|Number = Plur	‘they’
онѣма	онъ	Case = Dat|Gender = Masc|Number = Dual	‘them’
она	онъ	Case = Nom|Gender = Fem|Number = Sing	‘she’
онѣ	онъ	Case = Nom|Gender = Fem|Number = Dual	‘they’
оно	онъ	Case = Acc|Gender = Neut|Number = Sing	‘it’
онѣхъ	онъ	Case = Gen|Gender = Neut|Number = Plur	‘their’

**Table 5:** Example of English pronoun forms and lemmas as found in the UD.

Form	Lemma
they	they
them	they
she	she
they	they
it	it
their	they

To conclude this section, we find considerable evidence for agreement between complexity measures, in the sense that they rank languages similarly on the complexity scale. In fact, more than half of the significant Spearman correlations across the two tracks are positive. This effect seems particularly pronounced for measures assigned to the same domain. The strongest positive correlations stem from the domain of morphology, suggesting that here different researchers largely agree in their operationalization of complexity. This corroborates the result of an earlier meta-analysis (Berdicevskis et al. 2018), namely, that morphological complexity measures are more “robust” – in the sense of giving consistent results across different parts of UD treebanks – than syntactic complexity measures.

### 5.2 Trade-offs

We now approach the rest of significant Spearman correlations – which are negative – and hence indicate potential complexity trade-offs. In Track A, there are none after correction for multiple testing. In Track B, on the other hand, there are 14 out of 43 (33%) significant correlations which are negative.

In Track A, the highest negative correlations are found between the so-called *Syllable Information Density* (O_SID), on one hand, and Word Information Density (O_WID, described in detail above) as well as type-token-ratios (GM_TTR), on the other. The latter relationship is plotted in Figure 4 panel c. SID is defined – in parallel to WID – as the average ratio between the number of syllables per sentence in a baseline language (Korean is chosen in this case) and the respective target language.8See also the description by Yoon Mi Oh in the PBCtrack folder on github: https://github.com/IWMLC/language-complexity-metrics/. Note that the SID measure depends on automatic syllabification, which is currently only available for 10 languages of the original sample. For example, Finnish has relatively few word tokens, but relatively many syllable tokens per sentence, and hence ranks high in terms of TTR and WID, but low in terms of SID. On the other hand, English has many word tokens, but relatively few syllable tokens per sentence, and hence ranks low on TTR and WID, but high on SID. This reflects a genuine information-theoretic trade-off across languages: more information at the syllable level might counterbalance a lack of information at the word level. This is in line with earlier studies reporting negative correlations between the number of words per clause and syllables per word (Coloma 2017b, 2020; Fenk-Oczlon and Fenk 2005, 2008). Trade-offs between the complexity of word forms and internal word structure (Gutierrez-Vasques and Mijangos 2018) as well as phonotactic complexity and word length (Pimentel et al. 2020) also point in this direction. However, note that these negative correlations of Track A are not significant anymore after correction for multiple testing. This is certainly related to the low number of data points (i.e. only 10 for which syllabification is available).

In Track B, we find a multitude of negative correlations, in particular between measures across the two domains of syntax and morphology. This is visible in the upper left square of the correlogram (Figure 3): there are more blue (negative) cells than red (positive) cells (11/16 or 69%).

The strongest negative correlation – given in Figure 4 panel d – we find between the so-called Mean Feature Entropy (CR_mfe) and the average dependency length in possessive noun phrases (SI_dep_dl). The MFE is another measure of morphological complexity based on the distribution of inflectional features available in the UD data. It strongly positively correlates with measures discussed before, such as MSP and Inflectional Entropy, and we will not discuss it in more detail here. The SI_dep_dl measure, on the other hand, is defined as the “average dependency length in possessive NPs in which the relation between head and dependent is marked on the dependent.” To illustrate this, consider the English noun phrase examples in (3) and (4).9These examples are taken from the UD file English-EWT.conllu.

(3)

**Table:** 

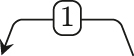	
his	**patience**

(4)

**Table:** 

	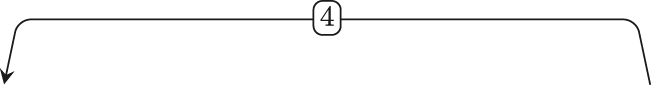	
the	company’s self-driven research	**strategy**

In the case of “his patience”, possession is marked on the dependent, i.e. on the pronoun (via a separate possessive form). Since the head and dependent are adjacent to one another, the dependency length is 1. In the latter example, however, there are three intervening words between the head (*strategy*) and the dependent (*company*), which gives us a dependency length of 4. The average dependency length is estimated to lie between ca. 2–3 across the English UD corpora, which is quite high compared to other languages.

Another language with high dependency lengths in this sense (ca. 3 on average) is Mandarin Chinese (cmn). In possessive NPs, it typically uses a separate possessive particle 的 ‘de’ which follows after the dependent being marked, and hence automatically increases the dependency length by +1. See the noun phrase example in (5).10Chinese examples are taken from the Chinese-GSD.conllu file of the UD corpus.

(5)

**Table:** 

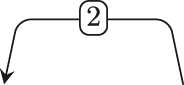		
我們 的 子孫		
wǒmen	de	**zǐsūn**
our	PART	offspring
“[…] our offspring […]”		

Just like in English, further words might intervene between the dependent and head. This is illustrated in example (6).

(6)

**Table:** 

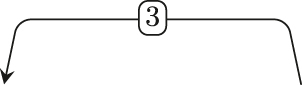			
他們 的 官方 聲明			
tāmen	de	guānfāng	**shēngmíng**
their(masc.)	PART	official	statement
“[…] their official statement […]”			

The relatively long dependencies in English and Mandarin Chinese contrast with languages for which the average lengths are estimated to around 1, meaning that possessor and possessee are virtually always directly adjacent to one another. Consider the examples from Classical Latin in (7)–(9).11These are taken from the Latin-PROIEL.conllu file of the UD corpus.

(7)

**Table:** 

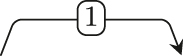	
**regnum** caelorum
regnum	caelorum
kingdom.NOM.SG	heaven.GEN.PL
“[…] kingdom of heavens […]”

(8)

**Table:** 

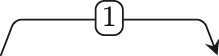	
**membrorum** tuorum
membrorum	tuorum
limb.GEN.PL	your.GEN.PL
“[…] your limbs […]”

(9)

**Table:** 

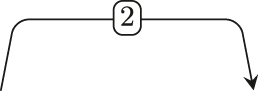		
**verbo**	autem	tuo
verbo	autem	tuo
word.ABL.SG	but	your.ABL.SG
“[…] but (from) your word […]”		

Possessive noun phrases like the ones in (7) and (8) are very common in the Latin corpus. That is, in the vast majority of cases, possessor and possessee occur in direct adjacency. In rare cases, we find another element intervening, e.g. the adverb *autem* ‘but/also’ in (9). Thus, the average dependency length is close to 1. This seems to be the case more generally in the languages which indicate possession by inflectional marking.

In a nutshell, the negative correlation between MFE and SI_dep_dl suggests that morphologically complex languages (e.g. Latin, Finnish, Old Church Slavonic, Basque) tend to have relatively short dependencies between possessor and possessee compared to less morphologically complex languages (e.g. English, Mandarin Chinese, and Vietnamese). Interestingly, this runs counter the common intuition that morphological marking allows for “scrambling” and displacement of words with mutual dependencies, which, as a consequence, would be expected to grow longer. In actual fact, dependencies become *shorter* in morphologically complex languages since there are fewer intervening elements (particles or other words) between head and dependent. Thus, the negative correlation between morphological complexity and dependency lengths could be seen as a genuine trade-off of the type expressed by Hockett (1958, 181): “what is not done morphologically has to be done syntactically.”

To conclude this section: We find that roughly one third (33%) of the significant Spearman correlations for pairs of measures are negative. Hence, there is considerable evidence for complexity trade-offs. Some of the strongest negative correlations concern information density at the syllable level compared to the word level (Track A), as well as an inverse relationship between average dependency lengths with the extend of inflectional marking (Track B).

### 5.3 Equi-complexity

In Section 2, we pointed out that finding negative correlations between complexity measurements is *per se* not sufficient to prove the equi-complexity hypothesis. On top of that, we have shown in the previous section that negative correlations – while certainly attested – are found alongside just as many (or more) positive correlations. Given this state of affairs, it might seem rather unlikely that the equi-complexity hypothesis will be met with strong statistical support. It is all the more surprising that, indeed, it is largely confirmed by the statistics.

**Figure 5:**
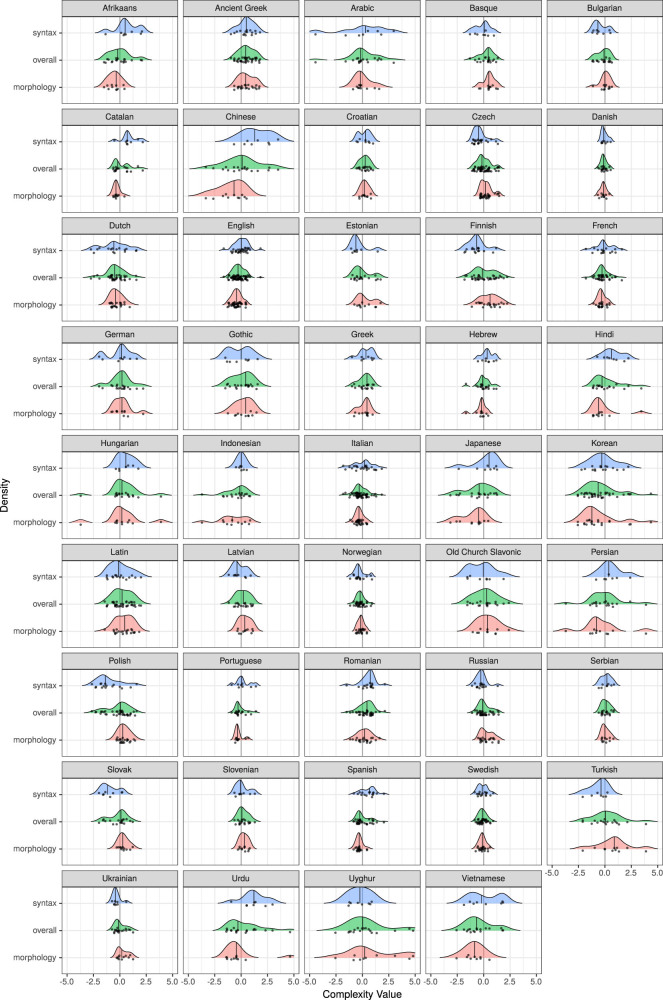
Density plot with measured complexity values for 44 languages of Track B (Universal Dependencies). Density distributions are given for the domain of morphology (red) and syntax (blue) separately, as well as for both combined, i.e. “overall” (green). Vertical black lines indicate median values. Values are centered and scaled to make them more commensurable.

In Figure 5, we can inspect the density distributions of complexity measurements across 44 languages. All the *p*-values and effect sizes for pairwise tests between languages are given in Appendix 5. Here we only discuss a few salient examples. Firstly, note that there are different types of languages. Some languages, for instance, Afrikaans, Chinese, Hindi, and Spanish, display a shift towards higher syntactic complexity paired with a shift towards lower morphological complexity. We find the opposite pattern in languages like Finnish, Latin, Latvian, Polish, and Turkish. A shift towards lower syntactic complexity is here paired with a shift towards higher morphological complexity. These visual observations are, in some cases, also backed up by the statistical results. For example, the effect size for the syntactic complexity shift between Chinese and Turkish is r=0.72 , and for the morphological complexity shift r=0.55 . In other words, there is statistical evidence that Mandarin Chinese is syntactically more complex than Turkish, but Turkish is morphologically more complex than Mandarin Chinese. So this is a typical example of how higher complexity in one domain might counterbalance lack of complexity in another. More generally, in both the domain of syntax and the domain of morphology, we find plenty of pairwise comparisons of languages where effect sizes are large, i.e., above 0.5.

Besides such “mirror images” of complexity, there are also further patterns to be observed. For instance, Danish is almost exactly at the center line for both syntax and morphology, while Ancient Greek is above for both, and Vietnamese is below for both. This might suggest that even when all measures are aggregated, i.e., in the “overall” complexity case, we could still find significant complexity shifts between languages. If we rank languages according to their median overall complexity value, we find Ancient Greek (0.43) at the top, and Vietnamese at the bottom (−0.66). However, this maximal difference between median values is neither associated with a significant *p*-value in the statistical test for a location shift (p>0.5) , nor with a large effect size for this test (r=0.39) . So, given the data and measurements provided by several researchers, we have no clear statistical support to claim that Ancient Greek is overall more complex than Vietnamese.

More generally, in all 946 pairwise tests for overall complexity shifts between these 44 languages, we find only five cases (0.5%) where p<0.05 . These five cases are: Ancient Greek/Dutch, Ancient Greek/English, Ancient Greek/Italian, Ancient Greek/Norwegian, and Romanian/English. Hence, there is a signal that Ancient Greek was overall more complex than (some) modern day Indo-European languages, though the effect sizes in all of these cases are rather medium (ca. 0.4). As a more general observation, there is not a single large effect size (>0.5) for the 946 tests on overall complexity shifts.

Now, it could be argued that, in a strict sense, the equi-complexity hypothesis is rejected if even just a single example of a significant location shift is found. However, since we here advocate a statistical account to address the given research questions, we would also advocate a “statistical mindset” when interpreting the results. Namely, if the research question is: how often and how forcefully can we reject the null hypothesis of equi-complexity? Then the statistical answer in our case is: very rarely and with low confidence.

Why do we find so few significant location shifts in the overall complexity vectors? – Complexity trade-offs are one part of the answer. As we discussed above, complexity shifts in one domain are often mirrored by adverse complexity shifts in another – though not necessarily. Another – maybe more important – reason is that the standard deviations of complexity vectors are often quite large, which impedes statistical significance for tests of location shifts. In other words, different measures often yield a wide range of values (even in the same domain and after standardization). This potentially has far reaching theoretical implications: we might increase representativity by including more metrics, but on the flip side, we decrease the certainty about the location of the overall complexity vector. This seems like Heisenberg’s *uncertainty principle* translated to measuring language complexity: we cannot be certain about representing all facets of a language’s complexity *and* be certain about the location of its complexity vector *at the same time*.

## 6 Open issues & caveats

Of course, the results presented here stand and fall with the corpora, language samples, and methods of analyses we choose. In the context of the IWMLC meeting, the Parallel Bible Corpus and the Universal Dependencies Corpora were selected due to their parallel nature (PBC), and their unified annotations (UD). On the downside, the texts in these corpora are relatively small in terms of number of tokens (in the tens of thousands). While standard measures in quantitative linguistics such as TTR and unigram entropy have been shown to stabilize for such sample sizes (Bentz et al. 2017a, 2017b), it is unclear if this is also the case for other measures which harness higher level annotations such as POS tags or dependency relations.

Likewise, given that there are currently more than 7,000 languages spoken and signed around the world, our overall sample of 80 languages only represents a small fraction of the actual linguistic diversity. The sample which was used for equi-complexity analyses only features 44 languages of which 31 are Indo-European. It also did not include Creole languages, which have been claimed to display the simplest overall grammars (McWhorter 2001). Since corpora such as the UD keep growing, including more and more non-Indo-European languages, our analyses can be re-assessed with larger language samples in the future.

On a more technical note, some measures hinge upon particular types of pre-processing, e.g. syllabification (SID) or tokenization into orthographic words (TTR and WID), which come with their own problems and caveats. For instance, TTR can be artificially high for scripts which do not delimit words by white spaces or other punctuation marks. This is the case for Burmese (mya) in Track A. It would typically not be considered a morphologically complex language, but ranks extremely high on both TTR and WID. Another technical problem is encountered with measures harnessing word forms and lemmas in the UD Track (e.g. inflectional entropy, MSP, and MFE from above). For instance, in the Korean corpora, what are supplied as “lemmas” are actually morphologically segmented word forms. This means there is a one-to-one correspondence between “form” and “lemma”, which brings down the values of some morphological complexity measures (e.g. inflectional entropy and MSP) to virtually 0.

For our statistical analyses we chose well-known and well-described statistics, i.e. Spearman correlations and Wilcoxon tests. An avenue for future research is to built more advanced models which take into account interdependencies between languages and measures (e.g. by research team). For instance, Coloma (2017a, 2017b, 2020 proposes to use partial correlation coefficients as well as simultaneous-equation regression methods to control for such interdependencies.

On a more general note, we have not included measures beyond syntax and morphology, i.e. phonology, semantics, pragmatics. Even if we did, measuring overall complexity might still turn out a “wild goose chase” (Deutscher 2009) given that there is potentially “hidden complexity” which is not surfacing in the type of data and methods we used here (Bisang 2015). For similar reasons the equi-complexity hypothesis (in a very general sense) might be considered genuinely non-falsifiable since “for every change in complexity it can be argued that there is another component, in some hitherto unknown domain of language structure, pragmatics, or culture, where the amount of complexity would be leveled out,” (Kusters 2003, 12). However, it is worth bearing in mind that the equi-complexity hypothesis as originally formulated by Hockett (1958) made reference only to the domains of morphology and syntax, not to a potentially unbounded number of domains.

## 7 Conclusions

We have here presented a meta-analysis of complexity measurements across 80 typologically diverse languages belonging to 34 families. Linking back to the questions formulated in the introduction, we aimed to assess the statistical evidence for *trade-offs* between measures, as well as for overall *equi-complexity*.

Across all measures we found evidence for agreement as well as for trade-offs. Namely, around two thirds of the significant correlations were positive, and one third was negative. This picture changes when we compare within-domain correlations with across-domain correlations. Measures in the same domain tend to positively correlate, while across domains there is a tendency for more negative correlations.

With regards to the equi-complexity hypothesis we find overwhelming support that – when complexity measures are aggregated and equally weighed – the complexity vectors across languages are virtually indistinguishable in terms of their location statistics. Only in a small fraction – i.e. 0.5% (!) – of the pairwise tests do we find statistically significant location shifts, though with small or medium effect sizes. These cases mainly involve Ancient Greek compared to modern day Indo-European languages. Of course, this is an interesting finding by itself.

We would thus argue that while languages certainly evolve into different directions when it comes to complexity in different domains, there seems to be a strong pressure to keep the average complexity in a close range. Languages as typologically diverse as Turkish and Mandarin Chinese, Finnish and English, Vietnamese and Old Church Slavonic turn out equally complex overall. We cannot help but marvel at this uniformity.

## Supplementary Material

Supplementary Material Details

Supplementary Material Details

Supplementary Material Details

Supplementary Material Details

Supplementary Material Details
