# Supplementary material for: Complexity trade-offs and equi-complexity in natural languages: a meta-analysis
Source: Linguist Vanguard. 2022 Oct 14;9(Suppl1):9–25. doi: 10.1515/lingvan-2021-0054 (PMC10234276; doi:10.1515/lingvan-2021-0054)
Supplement: Supplementary file 1 — Supplementary Material Details [file j_lingvan-2021-0054_suppl_001.pdf]

Appendix 1: Theoretical Background

Chris Bentz

July 20, 2022

Session Info

Give the session info (reduced).

```
## [1] "R version 3.6.3 (2020-02-29)"
```

```
## [1] "x86_64-pc-linux-gnu"
```

Load Libraries

If the libraries are not installed yet, you need to install them using, for example, the command: install.packages("ggplot2").

```
library(MASS)
library(ggplot2)
library(plyr)
library(GGally)
library(rstatix)
library(gridExtra)
```

Give the package versions.

```
## gridExtra   rstatix    GGally      plyr   ggplot2      MASS
##     "2.3"   "0.7.0"   "2.1.2"   "1.8.6"   "3.3.5"  "7.3-54"
```

Introduction

We here give a proof by counterexample that negative correlations between measurements of complexity in different domains (i.e. complexity trade-offs) do not strictly entail equi-complexity (in the sense of equality of mean complexity) of languages.

Theoretical Background

Let us assume we have n languages for which we measure complexities in two domains, e.g. syntax and morphology, such that we get two samples of measurements $m = (m_1, m_2, \ldots, m_n)$ and $s = (s_1, s_2, \ldots, s_n)$. This situation is illustrated in the table below.

language	m	s
L_1	m_1	s_1
L_2	m_2	s_2
L_3	m_3	s_3
L_4	m_4	s_4
L_5	m_5	s_5
...	...	...
L_n	m_n	s_n

Trade-offs as Negative Correlations

Trade-offs are here conceptualized as negative correlations. We here choose the Pearson (r) and Spearman (ρ) correlation coefficients as examples. While the former measures linear dependence, the latter is a non-parametric rank correlation.

Across the languages L_1 to L_n the Pearson correlation coefficient between the complexity measurements in the two domains (e.g. morphology and syntax) is defined as

$$r_{ms} = \frac{\sum_{i=1}^{n}(m_i - \overline{m})(s_i - \overline{s})}{\sqrt{\sum_{i=1}^{n}(m_i - \overline{m})^2}\sqrt{\sum_{i=1}^{n}(s_i - \overline{s})^2}}, \tag{1}$$

where n is the number of data points in the paired samples, m_i and s_i are individual measurements in the respective domain, and $\overline{m}$ and $\overline{s}$ are the arithmetic means of the samples (columns in the table above), i.e. complexity measurements in a certain domain, with

$$\overline{m} = \frac{1}{n}\sum_{i=1}^{n} m_i, \tag{2}$$

and

$$\overline{s} = \frac{1}{n}\sum_{i=1}^{n} s_i. \tag{3}$$

We have a negative correlation $r_{ms} < 0$ iff the numerator is negative, i.e.

$$\sum_{i=1}^{n}(m_i - \overline{m})(s_i - \overline{s}) < 0. \tag{4}$$

Note that the denominator cannot be negative.

The Spearman correlation coefficient, on the other hand, is defined as

$$\rho_{ms} = 1 - \frac{6\sum_{i=1}^{n}(\mathrm{rank}(m_i) - \mathrm{rank}(s_i))^2}{n(n^2-1)}, \tag{5}$$

where rank() is a function that gives the rank of the respective value when the values are ranked in ascending order (i.e. the smallest receives rank 1, the second smallest rank 2 etc.). Note that this definition only holds for distinct integers being ranked, which we will assume here for simplicity. We get a negative correlation iff

$$\frac{6\sum_{i=1}^{n}(\mathrm{rank}(m_i) - \mathrm{rank}(s_i))^2}{n(n^2-1)} > 1. \tag{6}$$

Equi-Complexity as Equality of Means

Furthermore, we conceptualize equi-complexity of languages L_1 to L_n here as equality of arithmetic means. For instance, if language L_1 has a morphological complexity of $m_1 = 5$ and a syntactic complexity of $s_1 = 1$, and language L_2 has $m_2 = 1$ and $s_2 = 5$, then the arithmetic mean complexity is 3 for both. Hence, they are considered overall equally complex. Assume more generally that m_j and s_j and m_k and s_k represent complexity measurements for two languages L_j and L_k. We would then consider the languages equi-complex iff

$$\overline{L_j} = \overline{L_k}, \tag{7}$$

where

$$\overline{L_j} = \frac{1}{2}(m_j + s_j), \tag{8}$$

and

$$\overline{L_k} = \frac{1}{2}(m_k + s_k). \tag{9}$$

For simplicity we here have just two different domains and just one measure per domain, i.e. one measure for morphology and one for syntax. Of course, in actual practice we could also have more than two domains, and several measures per domain.

Proof

We here proof by counterexample that neither a negative Pearson nor a negative Spearman correlation between two samples of complexity measurements in different domains strictly entails equality of means for the respective languages from which these measurements were taken. In other words, we will disproof by counterexample the claim that

$$(r_{ms} < 0), (\rho_{ms} < 0) \vdash (\overline{L_j} = \overline{L_k}). \tag{10}$$

Firstly, assume that we have a perfect negative Pearson and Spearman correlation between two samples, i.e. $r_{ms} = \rho_{ms} = -1$. For example, assume that the morphological complexities are m = (1, 2, 3, 4, 5) across five different languages, and the syntactic complexities can be perfectly linearly predicted by $s = 6 - 1m$. In other words, the syntactic complexity values are a linear transformation of the morphological complexity values. We thus have the following table of measurements per language and domain:

```
language = c("L1", "L2", "L3", "L4", "L5")
m <- c(1, 2, 3, 4, 5)
s <- 6 + -1*m
example.df <- data.frame(language, m, s)
print(example.df)
```

```
##   language m s
## 1       L1 1 5
## 2       L2 2 4
## 3       L3 3 3
## 4       L4 4 2
## 5       L5 5 1
```

This data set is visualized in the plot below.

```
plot(example.df$m, example.df$s, xlab = "morphological complexity (m)",
     ylab = "syntactic complexity (s)", xlim = c(1, 5.5))
text(example.df$m, example.df$s, labels = example.df$language, cex = 0.7, pos = 4)
```

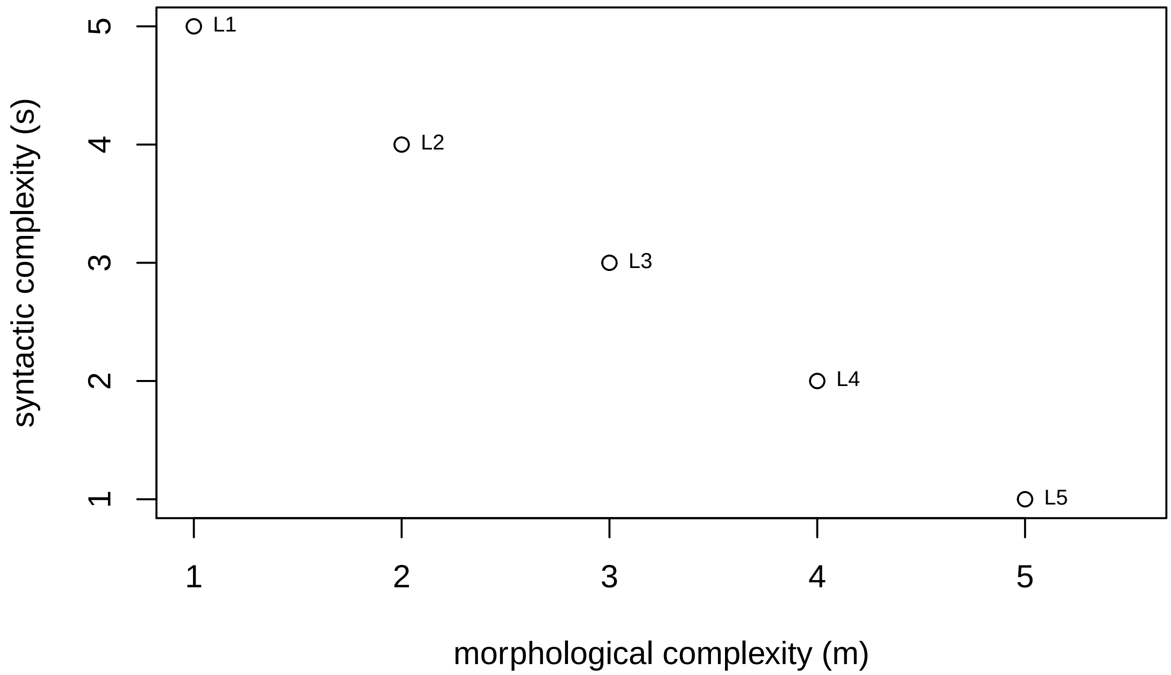

In this case, we indeed have equality of means across the languages, namely

$$\overline{L}_1 = \overline{L}_2 = \overline{L}_3 = \overline{L}_4 = \overline{L}_5 = 3. \tag{11}$$

Note that in this particular case, we also have $\overline{m} = \overline{s} = 3$. However, it is of course not necessarily the case that the means for languages and the means of measurements per domain are equal.

For the numerator of the Pearson correlation we have

$$\begin{aligned} \sum_{i=1}^{n}(m_i - \overline{m})(s_i - \overline{s}) = \\ (1-3)(5-3) + (2-3)(4-3) + (3-3)(3-3) + (4-3)(2-3) + (5-3)(1-3) = \\ -4 - 1 + 0 - 1 - 4 = -10. \end{aligned} \tag{12}$$

The condition for a negative Pearson correlation in equation (4) is hence fulfilled. In fact, in this particular case it is a perfect negative correlation ($r_{ms} = -1$) since the denominator of the formula for the Pearson coefficient in equation (1) evaluates to 10.

This result can be double-checked with the R function cor().

```
cor(example.df$m, example.df$s, method = "pearson")
```

```
## [1] -1
```

In order to evaluate the Spearman condition (equation (6)) for a negative correlation we might first create a table with the rankings of complexity measurements in ascending order. Note, however, that in our particular

case, the rankings coincide with the sample values themselves, i.e. the ranking for m is rank(m_1) = 1, rank(m_2) = 2, etc. Hence, we can simply take the original values as the output of rank().

$$\begin{aligned} \frac{6\sum_{i=1}^{n}(\text{rank}(m_i)-\text{rank}(s_i))^2}{n(n^2-1)} &= \\ \frac{6((1-5)^2+(2-4)^2+(3-3)^2+(4-2)^2+(5-1)^2)}{5(5^2-1)} &= \\ \frac{6((-4)^2+(-2)^2+(0)^2+(2)^2+(4)^2)}{5(24)} &= \\ \frac{240}{120} &= 2 \end{aligned} \tag{13}$$

We thus also get a perfect negative Spearman correlation of $\rho_{ms} = -1$.

This result can be double-checked with the R function cor().

```
cor(example.df$m, example.df$s, method = "spearman")
```

```
## [1] -1
```

To summarize, in our example data set we have perfect negative correlations between the measurements in the two domains, i.e. a perfect trade-off between morphological and syntactic complexity, and we have equality of means across the languages, i.e. overall equi-complexity.

Now, let us "flatten" the curve by decreasing the slope with which the syntactic complexity decreases with morphological complexity to 0.5 (instead of 1 as before), such that we have $s' = 5 - 0.5m$. We thus get the following syntactic complexity values:

```
language = c("L1", "L2", "L3", "L4", "L5")
m <- c(1, 2, 3, 4, 5)
s_prime1 <- 5 - 0.5*m
example.df <- data.frame(language, m, s, s_prime1)
print(example.df)
```

```
##   language m s s_prime1
## 1       L1 1 5      4.5
## 2       L2 2 4      4.0
## 3       L3 3 3      3.5
## 4       L4 4 2      3.0
## 5       L5 5 1      2.5
```

This data set is visualized in the plot below.

```
plot(example.df$m, example.df$s_prime1, xlab = "morphological complexity (m)",
     ylab = "syntactic complexity (s_prime)", xlim = c(1, 5.5), ylim = c(0, 5))
text(example.df$m, example.df$s_prime1, labels = example.df$language, cex = 0.7, pos = 4)
```

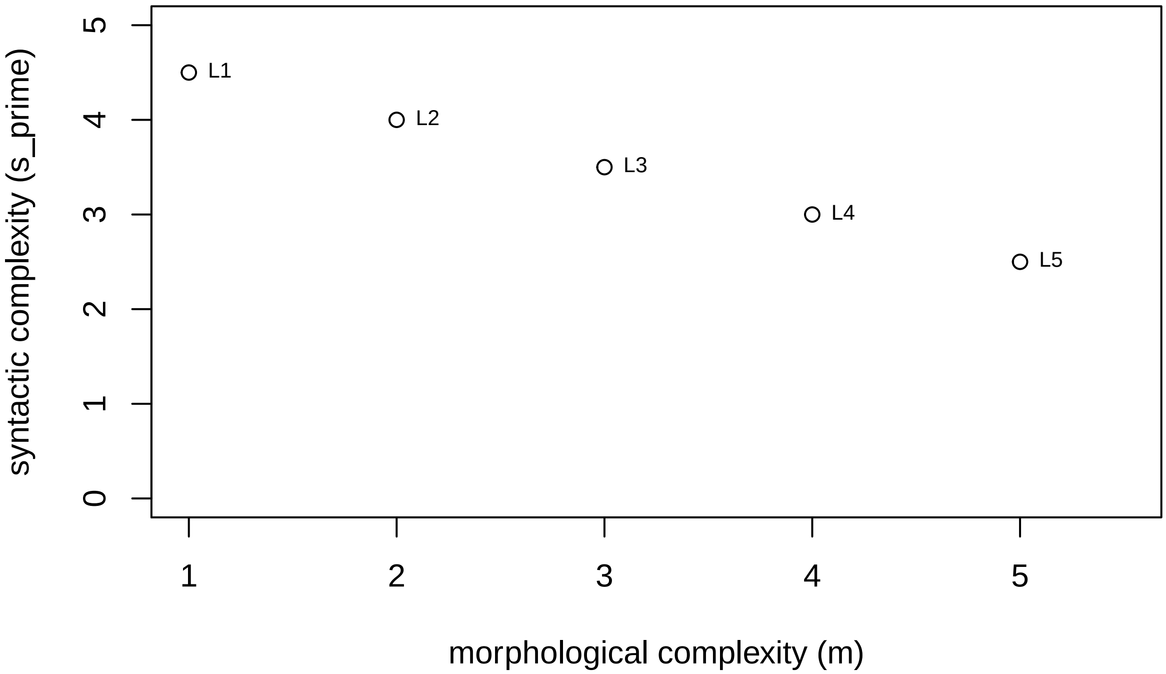

Since the syntactic complexity here decreases less than the morphological complexity increases, we now get differing mean values across the languages.

$$\begin{aligned} \overline{L_1'} &= \frac{4.5+1}{2} = 2.75 \\ \overline{L_2'} &= \frac{4+2}{2} = 3 \\ &\textit{etc.} \end{aligned} \tag{14}$$

For the condition for a negative Pearson correlation we now get

$$\begin{aligned} \sum_{i=1}^{n}(m_i - \overline{m})(s_i' - \overline{s}') = \\ (1-3)(4.5-3.5) + (2-3)(4-3.5) + (3-3)(3.5-3.5) + (4-3)(3-3.5) + (5-3)(2.5-3.5) = \\ -4 - 0.5 + 0 - 0.5 - 2 = -7. \end{aligned} \tag{15}$$

Hence, this condition is still fulfilled, and we have a negative Pearson correlation. In fact, the Pearson correlation is still perfect, i.e. -1, as can be double-checked with the cor() function.

```
cor(example.df$m, example.df$s_prime1, method = "pearson")
```

```
## [1] -1
```

In order to evaluate the Spearman condition (equation (6)), we first create a table with the rankings of complexity measurements in ascending order.

```
language = c("L1", "L2", "L3", "L4", "L5")
m <- c(1, 2, 3, 4, 5)
s <- 6 - 1*m
s_prime1 <- 5 - 0.5*m
```

```r
rank_m <- c(1, 2, 3, 4, 5)
rank_s_prime1 <- c(5, 4, 3, 2, 1)
example.df <- data.frame(language, m, s, s_prime1, rank_m, rank_s_prime1)
print(example.df)
```

```
##   language m s s_prime1 rank_m rank_s_prime1
## 1       L1 1 5      4.5      1             5
## 2       L2 2 4      4.0      2             4
## 3       L3 3 3      3.5      3             3
## 4       L4 4 2      3.0      4             2
## 5       L5 5 1      2.5      5             1
```

We can already see in the table that the rank order involving s' is equivalent to s. Hence, we get the exact same result as before.

$$\frac{6\sum_{i=1}^{n}(\text{rank}(m_i) - \text{rank}(s'_i))^2}{n(n^2-1)} = 2 \tag{16}$$

We thus also still have a perfect negative Spearman correlation.

This result can be double-checked with the R function cor().

```r
cor(example.df$m, example.df$s_prime1, method = "spearman")
```

```
## [1] -1
```

In sum, a linear transformation which "flattens" the curve between morphological and syntactic complexity values does not impact the Pearson or Spearman correlation, i.e. it preserves the trade-offs. However, it changes the mean values of languages, and hence breaks the equality of means.

Standardization

Standardization of linearly transformed data

In the proof above we have used two slightly differing linear transformations to derive s and s' from m. A general formula for linear transformations is $y = a + bx$. In the first case we used a = 6 and b = -1, and in the second case a = 5 and b = -0.5. It is noteworthy in this context that standardization of the sample values neutralizes the effect of linear transformations on the mean values of languages.

Standardization typically involves centering (which is defined as subtracting the mean value from all individual values of a sample), and scaling (which is defined as dividing the values of a sample by the standard deviation of the sample.). For instance, with the morphological complexity values centering is defined as

$$m^{\text{centered}} = m - \overline{m}, \tag{17}$$

and scaling is defined as

$$m^{\text{scaled}} = \frac{m}{\sigma^m}, \tag{18}$$

where σ^m is the standard deviation of the morphological complexity values.

Standardization then typically involves both centering and scaling, such that we have

$$m^{\text{standardized}} = \frac{m - \overline{m}}{\sigma^m}, \tag{19}$$

As an example, let us standardize the m, s, and s' sample values from above:

```
m_standardized <- (m-mean(m))/sd(m)
s_standardized <- (s-mean(s))/sd(s)
s_prime1_standardized <- (s_prime1-mean(s_prime1))/sd(s_prime1)
example.df <- data.frame(m, s, s_prime1, m_standardized, s_standardized, s_prime1_standardized)
example.df
```

```
##   m s s_prime1 m_standardized s_standardized s_prime1_standardized
## 1 1 5      4.5     -1.2649111      1.2649111             1.2649111
## 2 2 4      4.0     -0.6324555      0.6324555             0.6324555
## 3 3 3      3.5      0.0000000      0.0000000             0.0000000
## 4 4 2      3.0      0.6324555     -0.6324555            -0.6324555
## 5 5 1      2.5      1.2649111     -1.2649111            -1.2649111
```

Remember that each row of this data frame corresponds to values of a different language. As we have pointed out above, the mean values per language given m and s' are different, i.e.

```
(m + s_prime1)/2
```

```
## [1] 2.75 3.00 3.25 3.50 3.75
```

However, this effect of the linear transformation from s to s' on the mean values of the languages is neutralized if we standardize both m and s', i.e.

```
(m_standardized + s_prime1_standardized)/2
```

```
## [1] 0 0 0 0 0
```

Thus, due to standardization of the data, the mean values across languages are again equal, despite the linear transformation of s to s'.

Standardization of non-linearly transformed data

Apart from linear transformations we could also consider non-linear transformations of the data. For instance, by squaring the original syntactic complexity values such that we have

$$s'' = s^2. \tag{20}$$

With our example from above this would yield:

```
language = c("L1", "L2", "L3", "L4", "L5")
m <- c(1, 2, 3, 4, 5)
s <- 6 - 1*m
s_prime2 <- s^2
example.df <- data.frame(language, m, s, s_prime2)
print(example.df)
```

```
##   language m s s_prime2
## 1       L1 1 5       25
## 2       L2 2 4       16
## 3       L3 3 3        9
## 4       L4 4 2        4
## 5       L5 5 1        1
```

For the Pearson correlation we thus get:

```
cor(example.df$m, example.df$s_prime2, method = "pearson")
```

```
## [1] -0.9811049
```

and for the Spearman correlation we get:

```
cor(example.df$m, example.df$s_prime2, method = "spearman")
```

```
## [1] -1
```

As we would expect, the non-linear transformation impacts the Pearson correlation (at least slightly), but not the Spearman correlation. Moreover, just as in the case of a linear transformation discussed above, the mean values per language now differ, i.e.

```
(m + s_prime2)/2
```

```
## [1] 13  9  6  4  3
```

Importantly, note that in this case of a non-linear transformation, the inequality of means is preserved even under standardization.

```
m_standardized <- (m-mean(m))/sd(m)
s_prime2_standardized <- (s_prime2-mean(s_prime2))/sd(s_prime2)
# get correlations between standardized samples
cor(m_standardized, s_prime2_standardized, method = "pearson")
```

```
## [1] -0.9811049
```

```
cor(m_standardized, s_prime2_standardized, method = "spearman")
```

```
## [1] -1
```

```
# get mean values for standardized samples
(m_standardized + s_prime2_standardized)/2
```

```
## [1]  0.09146723 -0.05768392 -0.10341754 -0.04573362  0.11536784
```

Thus, standardization only recovers equality of means for the linear transformation we have discussed, but not for the non-linear transformation.

Combined Data and Plots

Combine all transformations and original data in one data frame for visualization purposes.

```
# original m and s values
language <- c("L1", "L2", "L3", "L4", "L5")
transformation <- rep("none", 5)
standardization <- rep("non-standardized", 5)
original.df <- data.frame(language, transformation, standardization, m, s)
colnames(original.df) <- c("language", "transformation",
                           "standardization", "morphology", "syntax")
# linear transformation of s
transformation <- rep("linear", 5)
linear.df <- data.frame(language, transformation, standardization, m, s_prime1)
colnames(linear.df) <- c("language", "transformation",
                         "standardization", "morphology", "syntax")
# non-linear transformation of s
transformation <- rep("non-linear", 5)
```

```r
nonlinear.df <- data.frame(language, transformation, standardization, m, s_prime2)
colnames(nonlinear.df) <- c("language", "transformation",
                            "standardization", "morphology", "syntax")
# standardized original
transformation <- rep("none", 5)
standardization <- rep("standardized", 5)
standardized.original.df <- data.frame(language, transformation,
                                       standardization, m_standardized, s_standardized)
colnames(standardized.original.df) <- c("language", "transformation",
                                        "standardization", "morphology", "syntax")
# standardized linear transformation of s
transformation <- rep("linear", 5)
standardization <- rep("standardized", 5)
standardized.linear.df <- data.frame(language, transformation,
                                       standardization, m_standardized,
                                     s_prime1_standardized)
colnames(standardized.linear.df) <- c("language", "transformation",
                                      "standardization", "morphology", "syntax")
# standardized non-linear transformation of s
transformation <- rep("non-linear", 5)
standardization <- rep("standardized", 5)
standardized.nonlinear.df <- data.frame(language, transformation,
                                        standardization, m_standardized,
                                        s_prime2_standardized)
colnames(standardized.nonlinear.df) <- c("language", "transformation",
                                         "standardization", "morphology", "syntax")
#combine all
combined.df <- rbind(original.df, linear.df, nonlinear.df, standardized.original.df,
                     standardized.linear.df, standardized.nonlinear.df)
# add mean values per language
combined.df$mean <- (combined.df$morphology + combined.df$syntax)/2
print(combined.df)
```

```
##    language transformation  standardization morphology     syntax        mean
## 1        L1           none non-standardized  1.0000000  5.0000000  3.00000000
## 2        L2           none non-standardized  2.0000000  4.0000000  3.00000000
## 3        L3           none non-standardized  3.0000000  3.0000000  3.00000000
## 4        L4           none non-standardized  4.0000000  2.0000000  3.00000000
## 5        L5           none non-standardized  5.0000000  1.0000000  3.00000000
## 6        L1         linear non-standardized  1.0000000  4.5000000  2.75000000
## 7        L2         linear non-standardized  2.0000000  4.0000000  3.00000000
## 8        L3         linear non-standardized  3.0000000  3.5000000  3.25000000
## 9        L4         linear non-standardized  4.0000000  3.0000000  3.50000000
## 10       L5         linear non-standardized  5.0000000  2.5000000  3.75000000
## 11       L1     non-linear non-standardized  1.0000000 25.0000000 13.00000000
## 12       L2     non-linear non-standardized  2.0000000 16.0000000  9.00000000
## 13       L3     non-linear non-standardized  3.0000000  9.0000000  6.00000000
## 14       L4     non-linear non-standardized  4.0000000  4.0000000  4.00000000
## 15       L5     non-linear non-standardized  5.0000000  1.0000000  3.00000000
## 16       L1           none     standardized -1.2649111  1.2649111  0.00000000
## 17       L2           none     standardized -0.6324555  0.6324555  0.00000000
## 18       L3           none     standardized  0.0000000  0.0000000  0.00000000
## 19       L4           none     standardized  0.6324555 -0.6324555  0.00000000
## 20       L5           none     standardized  1.2649111 -1.2649111  0.00000000
```

```
## 21       L1        linear    standardized -1.2649111  1.2649111  0.00000000
## 22       L2        linear    standardized -0.6324555  0.6324555  0.00000000
## 23       L3        linear    standardized  0.0000000  0.0000000  0.00000000
## 24       L4        linear    standardized  0.6324555 -0.6324555  0.00000000
## 25       L5        linear    standardized  1.2649111 -1.2649111  0.00000000
## 26       L1    non-linear    standardized -1.2649111  1.4478455  0.09146723
## 27       L2    non-linear    standardized -0.6324555  0.5170877 -0.05768392
## 28       L3    non-linear    standardized  0.0000000 -0.2068351 -0.10341754
## 29       L4    non-linear    standardized  0.6324555 -0.7239228 -0.04573362
## 30       L5    non-linear    standardized  1.2649111 -1.0341754  0.11536784
```

Scatterplot with original values.

```
scatterplot.original <- ggplot(combined.df[combined.df$standardization ==
                                             "non-standardized", ],
                      aes(x = morphology, y = syntax, group = transformation,
                          shape = language, color = transformation)) +
  geom_point(alpha = 0.7, size = 3) +
  geom_line() +
  ggtitle("a) Original Values") +
  xlab("Pseudo-Complexity Morphology") +
  ylab("Pseudo-Complexity Syntax") +
  theme(legend.position = "none")
#scatterplot.original
```

Scatterplot with standardized values.

```
scatterplot.standardized <- ggplot(combined.df[combined.df$standardization ==
                                                 "standardized", ],
                     aes(x = morphology, y = jitter(syntax), group = transformation,
                         shape = language, color = transformation)) +
  geom_point(alpha = 0.7, size = 3) +
  geom_line() +
  ggtitle("b) Standardized Values") +
  xlab("Pseudo-Complexity Morphology") +
  ylab("Pseudo-Complexity Syntax")
#scatterplot.standardized
```

Combine plots.

```
combined.plot <- grid.arrange(scatterplot.original, scatterplot.standardized,
                              ncol = 2, widths = c(1.5,2))
```

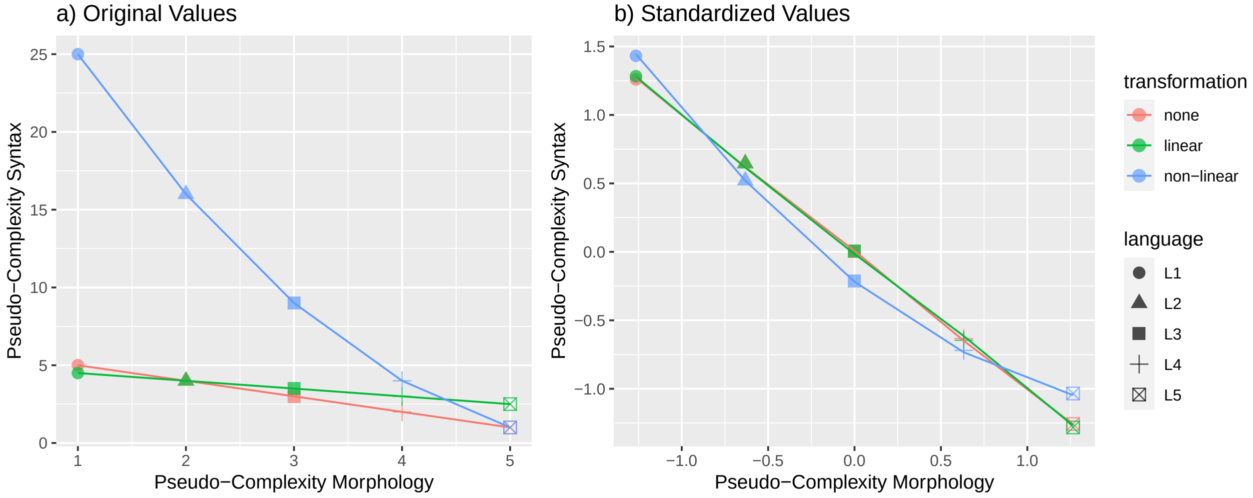

Save to file.

```
ggsave("Figures/Theoretical/transformationsPlot.pdf", combined.plot,
       dpi = 300, scale = 1, width = 9, height = 4, device = cairo_pdf)
```

Summary

In conclusion, measurements of complexity in separate domains (here morphology and syntax) might well have perfect Pearson and Spearman correlations across languages, while mean complexity values per language can still differ.

Firstly, we have illustrated this dissociation between negative correlations and equality of means by using a linear transformation of syntactic complexity measurements. This linear transformation preserves the Pearson and Spearman correlations across five languages, but breaks equality of means.

Secondly, we have shown that in the case of such a linear transformation, equality of means can be recovered by standardization of the values per domain. However, for non-linear transformations (at least the quadratic transformation discussed here) this is not necessarily the case.

This proofs that negative Pearson and Spearman correlations do not strictly entail equality of means, i.e.

$$(r_{ms} < 0), (\rho_{ms} < 0) \nvdash (\overline{L_j} = \overline{L_k}). \tag{21}$$

Open Issues

We have here given some analytic thoughts on the relationship between negative correlations and equality of means. However, we haven't considered the probabilistic component of random noise in the measurements, and statistical testing of equality of means. Also, we here only discussed the simple case of having one complexity measurement per domain, rather than several measurements. We address these points in a simulation study in a separate appendix.
