# Supplementary material for: Complexity trade-offs and equi-complexity in natural languages: a meta-analysis
Source: Linguist Vanguard. 2022 Oct 14;9(Suppl1):9–25. doi: 10.1515/lingvan-2021-0054 (PMC10234276; doi:10.1515/lingvan-2021-0054)
Supplement: Supplementary file 2 — Supplementary Material Details [file j_lingvan-2021-0054_suppl_002.pdf]

Appendix 2: Simulation Study

Chris Bentz

July 20, 2022

Session Info

Give the session info (reduced).

```
## [1] "R version 3.6.3 (2020-02-29)"
```

```
## [1] "x86_64-pc-linux-gnu"
```

Load Libraries

If the libraries are not installed yet, you need to install them using, for example, the command: install.packages("ggplot2").

```
library(MASS)
library(ggplot2)
library(plyr)
library(dplyr)
library(GGally)
library(rstatix)
```

Give the package versions.

```
##  rstatix   GGally    dplyr     plyr  ggplot2     MASS
##  "0.7.0"  "2.1.2"  "1.0.7"  "1.8.6"  "3.3.5" "7.3-54"
```

Introduction

The purpose of this file is to illustrate the decisions we take to statistically analyze the respective data, i.e. checking for normality of the data, choosing a statistical test for assessing equality in the mean values of two distributions, and getting effect sizes for this test. Both is achieved by using simulated data, i.e. pseudo-complexity measurements for two domains (e.g. syntax and morphology) across different languages. This also further illustrates the theoretical points made in the file on "theoretical background" with simulated data.

Generate Correlated Data

We here generate correlated pseudo-complexity measurements for for a given number of languages n. The number of measurements per domain can also be chosen (k). We then apply standardization, as well as linear and non-linear transformations to illustrate how this impacts the results of correlation and mean value analyses.

```
# set the seed for random number generation in order to
# get the same result when the code is re-run
set.seed(1)
# set parameters
n = 5 # number of languages
k = 10 # number of measures in domain
r = -0.9 # predefined correlation between the complexity values in the two domains
# generate the data
simulation.df <- data.frame()
for (i in 1:k){
  # generate two correlated samples
  data <- mvrnorm(n = n, mu = c(3, 3), Sigma = matrix(c(1, r, r, 1), nrow = 2),
                  empirical = TRUE)
  # original values
  value.m <- data[, 1]
  value.s <- data[, 2]
  value <- c(value.m, value.s)
  # scaled original values
  value.m.scaled <- scale(value.m)
  value.s.scaled <- scale(value.s)
  value.scaled <- c(value.m.scaled, value.s.scaled)
  # add information about domain and measurement id
  domain <- c(rep("morphology", n), rep("syntax", n))
  measure <- rep(i, n)
  # create vector with language names
  language <- c()
  for (j in 1:n){
    language <- c(language, paste("language", j))
  }
  local.df <- data.frame(language, measure, domain, value, value.scaled)
  # linear transformation to syntax of language 1
  local.df$value.lt <- local.df$value
  local.df$value.lt[local.df$language == "language 1" &
                    local.df$domain == "syntax"] <-
  local.df$value.lt[local.df$language == "language 1" &
                    local.df$domain == "syntax"]*0.5
  # scale the data
  local.df$value.lt.scaled <-
    c(value.m.scaled, scale(local.df$value.lt[local.df$domain == "syntax"]))
  # non-linear transformation to syntax of language 2
  local.df$value.nt <- local.df$value
  local.df$value.nt[local.df$language == "language 2" &
                    local.df$domain == "syntax"] <-
  local.df$value.nt[local.df$language == "language 2" &
                    local.df$domain == "syntax"]^1.5
  # scale the data
  local.df$value.nt.scaled <-
    c(value.m.scaled, scale(local.df$value.nt[local.df$domain == "syntax"]))
  # bind to overall data frame
  simulation.df <- rbind(simulation.df, local.df)
}
```

Put into wide format (for scatterplots below).

```r
# wide format
simulation.df.wide <- reshape(simulation.df, idvar = c("language", "measure"),
                              timevar = "domain", direction = "wide")
# select only certain columns for plotting
simulation.df.short <- select(simulation.df.wide, value.morphology,
                              value.scaled.morphology, value.lt.syntax, value.nt.syntax,
                              value.scaled.syntax, value.lt.scaled.syntax,
                              value.nt.scaled.syntax)
```

Scatterplot with Pearson Correlations

For all the data

```r
simulation.scatterplot <- ggpairs(simulation.df.short,
                        lower = list(continuous = wrap("smooth_loess", alpha = 0.3,
                                                       lwd = 0.5, size = 2)),
                        upper = list(continuous = wrap('cor', method = "pearson"))) +
                        theme_bw() +
                        theme(axis.text.x = element_text(angle = 90, hjust = 1))
print(simulation.scatterplot)
```

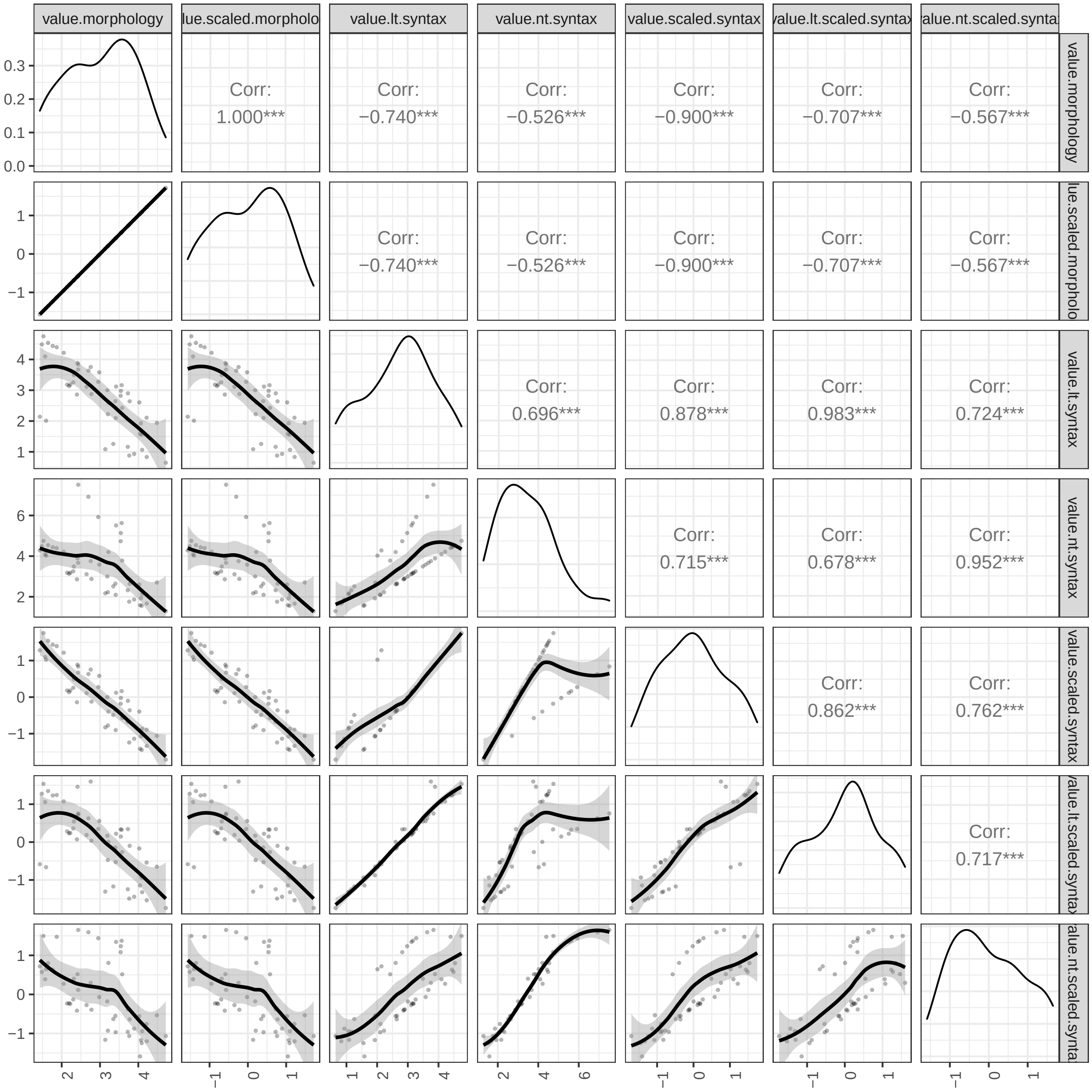

For selected parts of the data

Select the scaled morphological pseudo-complexity values, and the scaled syntactic pseudo-complexity values after non-linear transformation of the values of language 2. Note that for this data set we expect to find negative correlations *and* significant shifts in overall complexity vectors according to the proof in the appendix on the theoretical background.

```
scatterplot.selected <- ggplot(simulation.df.wide, aes(x = value.scaled.morphology,
                                                       y = value.nt.scaled.syntax,
                                                       color = language)) +
  geom_point(alpha = 0.7) +
  geom_smooth(method = lm, alpha = 0.1) +
  ggtitle("Pearson r = -0.57; Spearman rho = -0.62") +
```

```
  xlab("Pseudo-Complexity Morphology (Scaled)") +
  ylab("Pseudo-Complexity Syntax (Scaled)")
scatterplot.selected
```

```
## `geom_smooth()` using formula 'y ~ x'
```

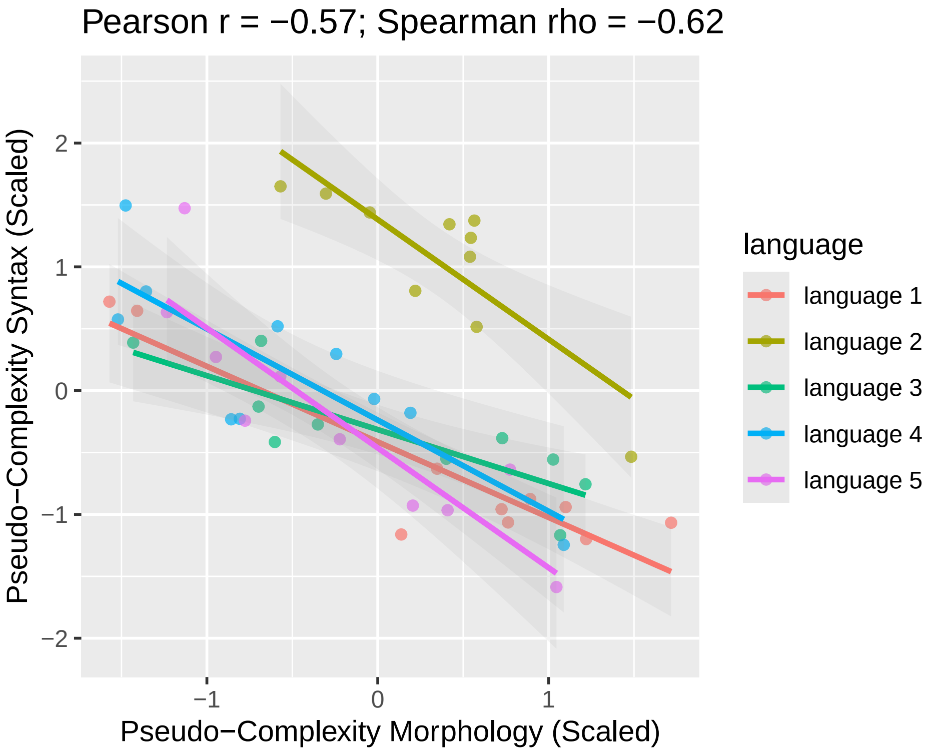

Density Distributions

Plot density distributions of complexity pseudo-measurements by language. Individual values for each complexity pseudo-measurement are plotted as black dots. The central value (0) is indicated by a vertical dotted line for visual reference. The median and mean values of complexity pseudo-measurements per language might also be indicated.

Standardized Samples

Get mean, median, and standard deviation values.

```
# get mean values for each language
mu <- ddply(simulation.df, "language", summarise,
            grp.mean = mean(value.scaled, na.rm = T))
# get median values for each language
med <- ddply(simulation.df, "language", summarise,
             grp.median = median(value.scaled, na.rm = T))
# get standard deviation values for each language
sdev <- ddply(simulation.df, "language", summarise,
              grp.sd = sd(value.scaled, na.rm = T))
```

Plot density distributions with indication of median (mean) values.

```
density.plot <- ggplot(simulation.df, aes(x = value.scaled,
                                          color = domain, fill = domain)) +
  geom_density(alpha = .2) +
  # add some jitter to prevent overplotting
  geom_jitter(data = simulation.df, aes(x = value.scaled, y = 0),
           size = 1, height = 0.001, width = 0) +
  geom_vline(aes(xintercept = 0), color = "darkgrey") +
  geom_vline(data = med, aes(xintercept = grp.median), linetype = "dashed") +
  facet_wrap(~ language) +
  #xlim(-3, 3) +
  labs(x = "Complexity Value", y = "Density") +
  theme_bw()
print(density.plot)
```

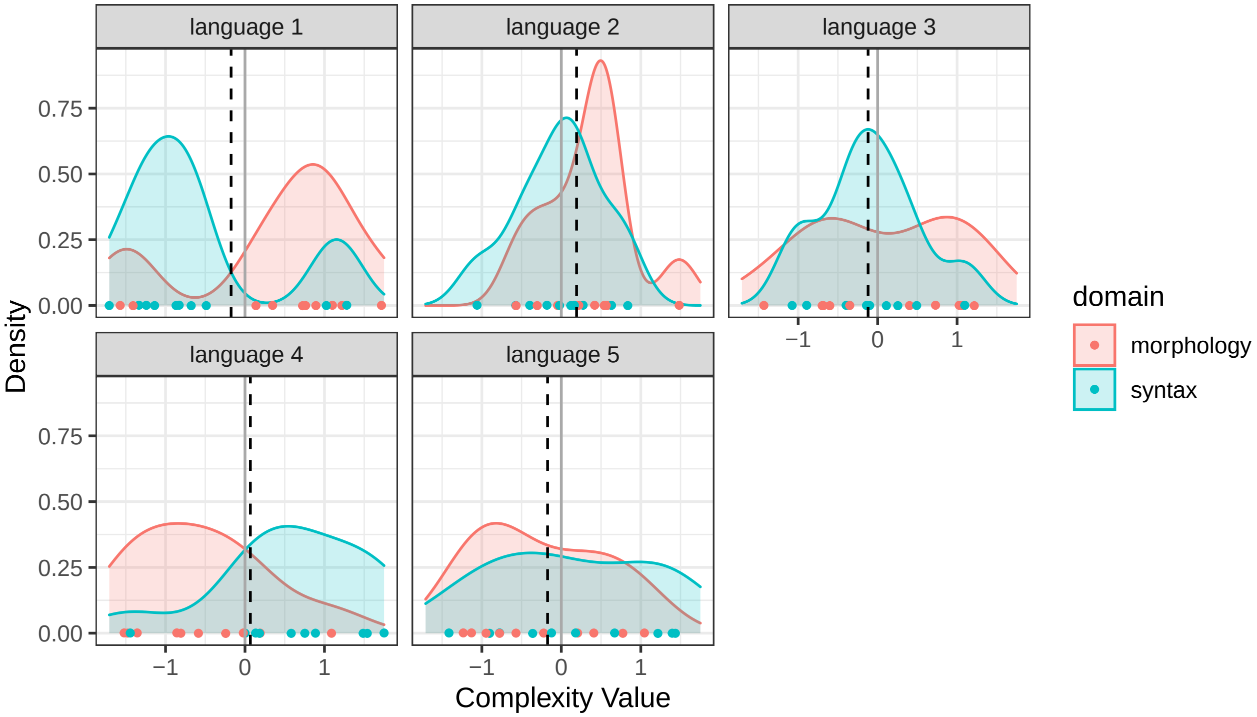

Standardized Samples with Non-Linear Transformation of Syntax Values

Get mean, median, and standard deviation values.

```
# get mean values for each language
mu <- ddply(simulation.df, "language", summarise,
            grp.mean = mean(value.nt.scaled, na.rm = T))
# get median values for each language
med <- ddply(simulation.df, "language", summarise,
             grp.median = median(value.nt.scaled, na.rm = T))
# get standard deviation values for each language
sdev <- ddply(simulation.df, "language", summarise,
              grp.sd = sd(value.nt.scaled, na.rm = T))
```

Plot density distributions with indication of median (mean) values.

```r
density.plot <- ggplot(simulation.df, aes(x = value.nt.scaled,
                                          color = domain, fill = domain)) +
  geom_density(alpha = .2) +
  # add some jitter to prevent overplotting
  geom_jitter(data = simulation.df, aes(x = value.nt.scaled, y = 0),
              size = 1, height = 0.001, width = 0) +
  geom_vline(aes(xintercept = 0), color = "darkgrey") +
  geom_vline(data = med, aes(xintercept = grp.median), linetype = "dashed") +
  facet_wrap(~ language) +
  #xlim(-3, 3) +
  labs(x = "Complexity Value", y = "Density") +
  theme_bw()
print(density.plot)
```

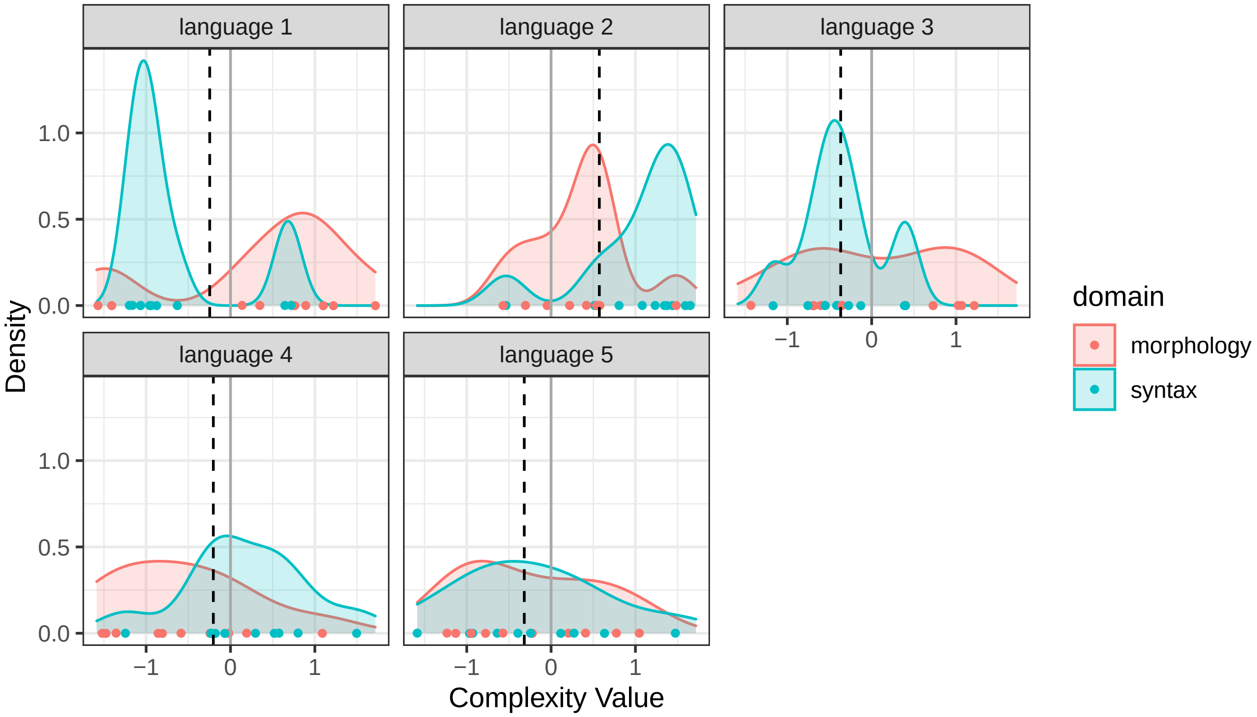

Normality

Check for normality of the data via QQ-plots. The dots should roughly follow a straight line (Crawley 2007, p. 281).

```r
# standardized samples
ggplot(simulation.df, aes(sample = value.scaled)) +
  stat_qq()
```

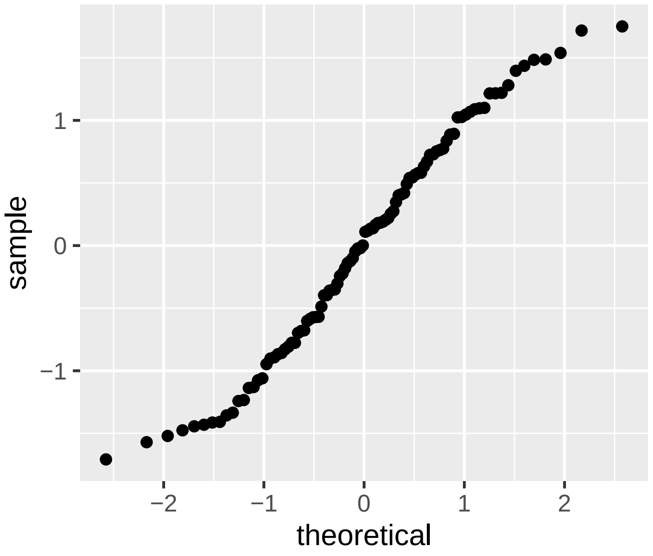


```
# standardized samples with non-linear transformation of syntax values
ggplot(simulation.df, aes(sample = value.nt.scaled)) +
  stat_qq()
```

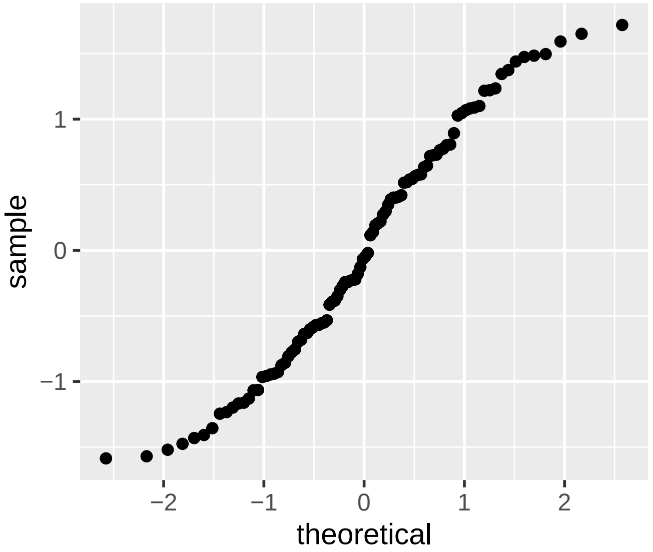

Choose statistical tests

Select a statistical test: We choose the Wilcoxon signed rank test here as a non-parametric alternative to the t-test (Crawley 2007, p. 283; Baayen 2008, p. 77). The data is "paired" here since we generated pseudo-complexity measures for all languages and domains.

Run pairwise Wilcoxon tests for the standardized original values.

```
# we add some random noise here to the value vector with
# the function jitter(), since we otherwise get warnings due to ties in the data
p.values <- pairwise.wilcox.test(jitter(simulation.df$value.scaled),
                                 simulation.df$language, paired = T,
                                 p.adjust.method = "holm")
p.values$p.value
```

```
##            language 1 language 2 language 3 language 4
## language 2          1         NA         NA         NA
## language 3          1          1         NA         NA
## language 4          1          1          1         NA
## language 5          1          1          1          1
```

Run pairwise Wilcoxon tests for the standardized values with non-linear transformation of syntactic values.

```
p.values <- pairwise.wilcox.test(jitter(simulation.df$value.nt.scaled),
                                 simulation.df$language, paired = T,
                                 p.adjust.method = "holm")
p.values$p.value
```

```
##            language 1 language 2 language 3 language 4
## language 2  0.1864929         NA         NA         NA
## language 3  1.0000000 0.06646729         NA         NA
## language 4  1.0000000 0.01016617          1         NA
## language 5  1.0000000 0.02092552          1          1
```

Effect Size

An overview of effect size measures per statistical test is given in Patil (2020). In conjunction with the Wilcoxon test we use the statistic r (i.e. function wilcox_effsize() of the "rstatix" package).

```
# standardized original
effect.sizes <- wilcox_effsize(simulation.df, value.scaled ~ language, paired = T)
# standardized with non-linear transformation of syntactic values
effect.sizes.nt <- wilcox_effsize(simulation.df, value.nt.scaled ~ language, paired = T)
```

Effect Size Heatmap

Plot heatmaps with effect sizes to get a better overview.

Standardized

```
effect.sizes.plot <- ggplot(as.data.frame(effect.sizes), aes(group1, group2)) +
  geom_tile(aes(fill = effsize), color = "white") +
  scale_fill_gradient2(low = "light blue", mid = "light grey", high = "red",
                       midpoint = 0.5, limit = c(0,1)) +
  geom_text(aes(label = round(effsize, 2))) +
  labs(x = "", y = "") +
  theme_minimal() +
  theme(axis.text.x = element_text(angle = 45, hjust = 1))
effect.sizes.plot
```

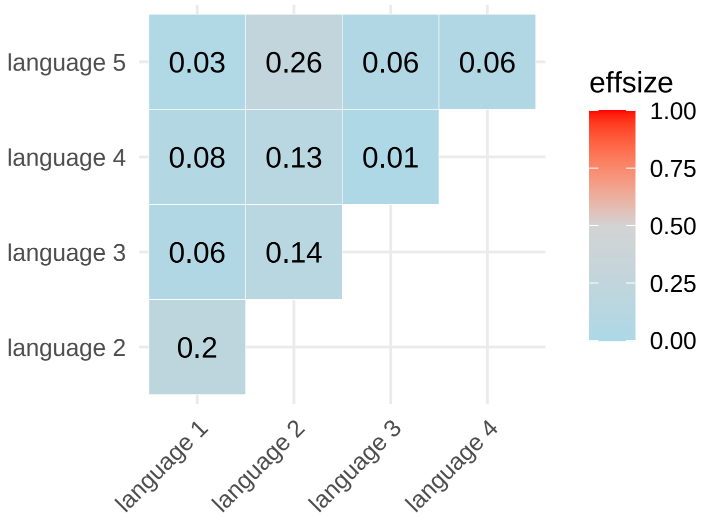

Standardized with non-linear transformation of syntactic values

```
effect.sizes.nt.plot <- ggplot(as.data.frame(effect.sizes.nt), aes(group1, group2)) +
  geom_tile(aes(fill = effsize), color = "white") +
  scale_fill_gradient2(low = "light blue", mid = "light grey", high = "red",
                       midpoint = 0.5, limit = c(0,1)) +
  geom_text(aes(label = round(effsize, 2))) +
  labs(x = "", y = "") +
  theme_minimal() +
  theme(axis.text.x = element_text(angle = 45, hjust = 1))
effect.sizes.nt.plot
```

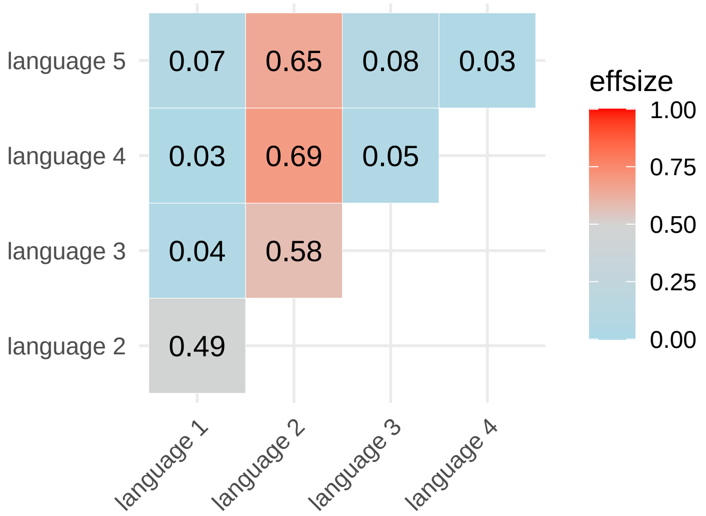

Interpretation

Firstly, we can see in the correlation plots that we consistently find negative and significant Pearson and Spearman correlations between morphological and syntactic pseudo-complexity values across languages.

Notice that for the standardized original data we also find no significant differences between any pair of languages in the Wilcoxon tests (p=1 for all pairs), and the effect sizes are in the range 0.01 to 0.26, i.e. small. However, for the data where we have a non-linear transformation of the syntactic pseudo-complexity values in language 2, we now find some significant differences in the Wilcoxon test (i.e. $p < 0.05$ for comparisons of language 2 with language 4 and 5), and these are matched with large effect sizes (i.e. 0.69 and 0.65).

This simulation hence corroborates the points of the proof in the appendix on "theoretical background' '. Namely, for standardized data with no transformations (or linear transformations) the link between negative correlations and equality of overall complexity holds. However, for data with non-linear transformations of pseudo-complexity values (here for language 2) this link breaks: we are able to detect significant shifts in the overall complexity location of language 2 despite the negative correlations in the domains.

References

Baayen, R. H. (2008). Analyzing linguistic data: A practical introduction using statistics in R. Cambridge University Press.

Crawley, M. J. (2007). The R book. John Wiley & Sons Ltd.

Patil, I. (2020). Test and effect size details. online at https://cran.r-project.org/web/packages/statsExpressions/vignettes/stats_details.html.
