# Supplementary material for: Complexity trade-offs and equi-complexity in natural languages: a meta-analysis
Source: Linguist Vanguard. 2022 Oct 14;9(Suppl1):9–25. doi: 10.1515/lingvan-2021-0054 (PMC10234276; doi:10.1515/lingvan-2021-0054)
Supplement: Supplementary file 3 — Supplementary Material Details [file j_lingvan-2021-0054_suppl_003.pdf]

Appendix 3: World Maps

Chris Bentz

July 20, 2022

Session Info

Give the session info (reduced).

```
## [1] "R version 3.6.3 (2020-02-29)"
```

```
## [1] "x86_64-pc-linux-gnu"
```

Load Libraries

If the libraries are not installed yet, you need to install them using, for example, the command: install.packages("ggplot2").

```
library(readr)
library(ggmap)
library(maps)
library(gridExtra)
library(ggrepel)
```

Give the package versions.

```
##   ggrepel gridExtra      maps     ggmap   ggplot2     readr
##   "0.9.1"     "2.3"   "3.4.0"   "3.0.0"   "3.3.5"   "2.0.2"
```

Load the Data

Load Glottolog (Version 4.1) language information combined with information on the language sample of the IWMLC.

```
languages <- as.data.frame(read_csv("https://raw.githubusercontent.com/IWMLC/language-complexity-metrics
```

Simple Stats

```
length(unique(languages$isocodes)) # number of languages according to iso
```

```
## [1] 80
```

```
length(unique(languages$glottocode)) # number of languages according to glottolog
```

```
## [1] 80
```

```r
length(unique(languages$family_id)) # number of language families according to glottolog
```

```
## [1] 34
```

```r
unique(languages$macroarea) # number of macroareas according to glottolog
```

```
## [1] "Africa"        "Papunesia"     "Eurasia"       "South America"
## [5] "North America" "Australia"     NA
```

Pre-Processing

```r
# remove Norwegian Nynorsk (nno) since this has NAs in glottolog
languages <- languages[languages$isocodes != "nno", ]
# add 360 to longitudes for languages with longitudes < -25
# (this is necessary to create a Pacific centered map)
languages$longitude[languages$longitude < -25] <-
  languages$longitude[languages$longitude < -25] + 360
```

World Map

World maps with macroarea information from Glottolog.

```r
# create world map
world <- map_data("world", wrap = c(-25, 335))
area.map <- ggplot() +
  geom_polygon(data = world, aes(x = long, y = lat, group = group),
               fill = "white", colour = "darkgrey") +
  geom_point(data = languages, aes(x = longitude, y = latitude,
                                   fill = macroarea),
             alpha = 0.9, size = 2.5, pch = 21) +
  geom_text_repel(data = languages, aes(x = longitude, y = latitude,
                                        label = name), size = 2.5,
                  box.padding = unit(0.1, 'lines'), force = 0.2) +
  scale_y_continuous(limits = c(-65, 80)) +
  labs(x = "longitude", y = "latitude", fill = "Macroarea") +
  theme_bw() +
  facet_wrap (~ corpus, nrow = 2) +
  theme(axis.title.x = element_text(size = 12),
        axis.title.y = element_text(size = 12),
        title = element_text(size = 12),
        legend.title = element_text(size = 10),
        legend.text = element_text(size = 10),
        legend.position = "bottom")
area.map
```

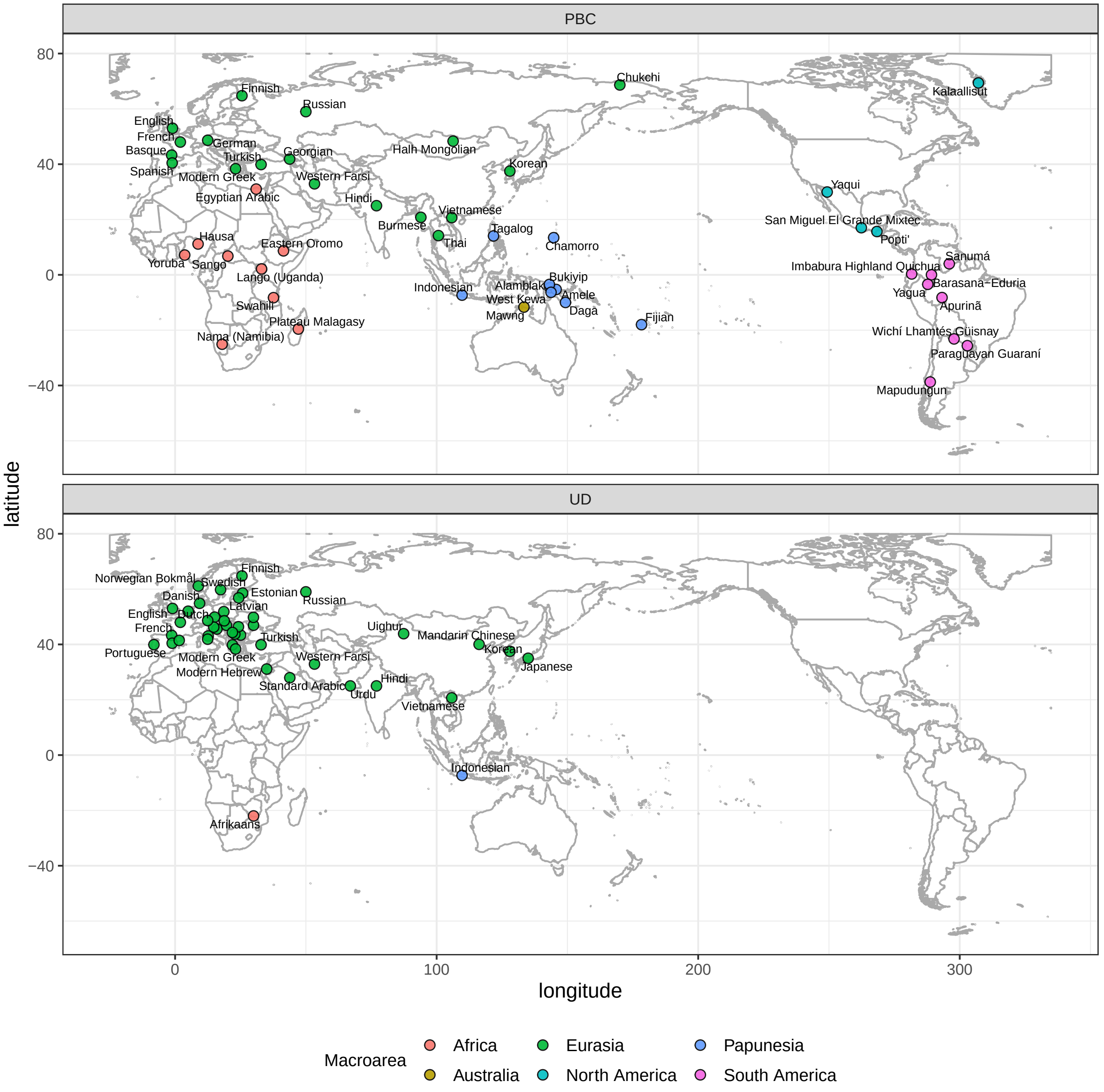

Save to file.

```
ggsave("Figures/WorldMap/worldMaps.pdf", area.map,
       dpi = 300, scale = 1, width = 9, height = 9, device = cairo_pdf)
```
