# Supplementary material for: Complexity trade-offs and equi-complexity in natural languages: a meta-analysis
Source: Linguist Vanguard. 2022 Oct 14;9(Suppl1):9–25. doi: 10.1515/lingvan-2021-0054 (PMC10234276; doi:10.1515/lingvan-2021-0054)
Supplement: Supplementary file 4 — Supplementary Material Details [file j_lingvan-2021-0054_suppl_004.pdf]

Appendix 4: Correlations Between Complexity Measures

Chris Bentz

July 20, 2022

Session Info

Give the session info (reduced).

```
## [1] "R version 3.6.3 (2020-02-29)"
## [1] "x86_64-pc-linux-gnu"
```

Load Libraries

If the libraries are not installed yet, you need to install them using, for example, the command: install.packages("ggplot2").

```
library(readr)
library(ggplot2)
library(gridExtra)
library(GGally)
library(ggrepel)
library(psych)
library(ggcorrplot)
```

Give the package versions.

```
## ggcorrplot      psych    ggrepel     GGally  gridExtra    ggplot2      readr
##    "0.1.3"    "2.1.9"    "0.9.1"    "2.1.2"      "2.3"    "3.3.5"    "2.0.2"
```

Introduction

This file gives the code to calculate correlations between empirical complexity measurements in Track A and Track B.

Load the Data

The participants' results are loaded as csv files directly from the github repository into separate data frames. We only use the name of the first author (lower case) to name the data frame.

```
#Track A (Parallel Bible Corpus, PBC)
gutierrez.results <- read_csv("https://raw.githubusercontent.com/IWMLC/language-complexity-metrics/maste
# remove the parentheses in column names
```

```
colnames(gutierrez.results) <- sub("\\(", "", colnames(gutierrez.results))
colnames(gutierrez.results) <- sub("\\)", "", colnames(gutierrez.results))
# replace "+" by "."
colnames(gutierrez.results) <- gsub("\\+", ".", colnames(gutierrez.results))
oh.results <- read_csv("https://raw.githubusercontent.com/IWMLC/language-complexity-metrics/master/PBCt

#TRACK B (Universal Dependencies, UD)
brunato.results <- read_csv("https://raw.githubusercontent.com/IWMLC/language-complexity-metrics/master/
coltekin.results <- read_csv("https://raw.githubusercontent.com/IWMLC/language-complexity-metrics/maste
semenuks.results <- read_csv("https://raw.githubusercontent.com/IWMLC/language-complexity-metrics/maste
sinnemaki.results <- read_csv("https://raw.githubusercontent.com/IWMLC/language-complexity-metrics/mast
sozinova.results <- read_csv("https://raw.githubusercontent.com/IWMLC/language-complexity-metrics/maste
```

Sanity check, look at the number of rows and columns of the data frames.

```
#Track A (should be 49 rows)
track.a.rows <- c(nrow(gutierrez.results), nrow(oh.results))
print(track.a.rows) # this corresponds to the number of languages
```

```
## [1] 49 49
```

```
track.a.cols <- c(ncol(gutierrez.results)-2, ncol(oh.results)-2)
print(track.a.cols) # this is the number of measures per team
```

```
## [1] 12  3
```

```
#Track B (should be 63 rows)
track.b.rows <- c(nrow(brunato.results), nrow(coltekin.results),
                  nrow(semenuks.results), nrow(sinnemaki.results),
                  nrow(sozinova.results))
print(track.b.rows) # this corresponds to the number of languages
```

```
## [1] 63 63 63 63 63
```

```
track.b.cols <- c(ncol(brunato.results)-2, ncol(coltekin.results)-2,
                  ncol(semenuks.results)-2, ncol(sinnemaki.results)-2,
                  ncol(sozinova.results)-2)
print(track.b.cols) # this is the number of measures per team
```

```
## [1] 11  6  2  6  2
```

Preprocessing

Put data into a single data frame.

```
track.a <- cbind(gutierrez.results, oh.results[, 3:ncol(oh.results)])
track.b <- cbind(brunato.results, coltekin.results[, 3:ncol(coltekin.results)],
                 semenuks.results[, 3:ncol(semenuks.results)],
                 sinnemaki.results[, 3:ncol(sinnemaki.results)],
                 sozinova.results[, 3:ncol(sozinova.results)])
```

Remove certain measures. To include all measures, this code can just be commented out. Note, however, that there are certain measures in Track A which are redundant in the sense that they only differ in whether the Bible texts are fully parallelized or not. In Track B, some measures given by the same team have strong positive correlations, e.g. the number of tokens in a sentence (BV_n_tokens) and the average number of tokens per clause (BV_avg_token_per_clause). We hence just keep one of the strongly correlated measures

to not inflate the number of correlated data points. Also there are measures with many NAs in Track B, i.e. "SI_double_dl", "SI_head_dl", "SI_zero_dl", which are removed here.

```
# Remove measures in Track A
track.a <- track.a[ , -which(names(track.a) %in% c("GM_H1gram", "GM_H3gram", "GM_TTR",
                                                   "GM_TTR.H1", "GM_TTR.H3", "GM_TTR.H1.H3",
                                                   "GM_TTR.H1_fullyparallelised",
                                                   "GM_TTR.H3_fullyparallelised",
                                                   "GM_TTR.H1.H3_fullyparallelised"))]
# Remove measures in Track B
track.b <- track.b[ , -which(names(track.b) %in% c("BV_avg_max_depth",
                                                   "BV_avg_token_per_clause",
                                                   "SI_double_dl", "SI_head_dl",
                                                   "SI_zero_dl"))]
```

Invert the values (by subtracting them from 1) for the measure "CR_inflection_accuracy" in Track B. Note that higher values in the original measure mean *lower* rather than higher complexity.

```
track.b$CR_inflection_accuracy <- 1-track.b$CR_inflection_accuracy
```

Center and scale all numerical columns to make them more comparable.

```
# keep meta-information columns again
track.a.scaled <- cbind(track.a[1:2], scale(track.a[3:ncol(track.a)]))
track.b.scaled <- cbind(track.b[1:2], scale(track.b[3:ncol(track.b)]))
```

Check the first 6 rows of the data.

```
#head(track.a.scaled)
#head(track.b.scaled)
```

Subset Track B data into Indo-European and non-Indo-European languages (these are used for separate correlational analyses below).

```
# select Indo-European languages
selection.IE <- c("Afrikaans", "Bulgarian", "Catalan", "Czech", "Old Church Slavonic",
                  "Danish", "German", "Greek", "English", "Persian",
                  "French", "Gothic", "Ancient Greek", "Hindi", "Croatian", "Italian",
                  "Latin", "Latvian", "Dutch", "Norwegian", "Polish", "Portuguese",
                  "Romanian", "Russian", "Slovak", "Slovenian", "Spanish", "Serbian",
                  "Swedish", "Ukrainian", "Urdu")
track.b.scaled.IE <- track.b.scaled[track.b.scaled$language %in% selection.IE, ]
# select non-Indo-European languages
selection.nonIE <- c("Arabic", "Chinese", "Estonian", "Basque", "Finnish", "Hebrew",
                     "Hungarian", "Indonesian", "Japanese", "Korean", "Turkish",
                     "Uyghur", "Vietnamese")
track.b.scaled.nonIE <- track.b.scaled[track.b.scaled$language %in% selection.nonIE, ]
```

Remove the first two columns of data frames (useful for plotting).

```
track.a.short <- track.a.scaled[, 3:ncol(track.a)]
track.b.short <- track.b.scaled[, 3:ncol(track.b)]
track.b.IE.short <- track.b.scaled.IE[, 3:ncol(track.b)]
track.b.nonIE.short <- track.b.scaled.nonIE[, 3:ncol(track.b)]
```

Scatterplots by Track

TRACK A

For visual reference, we here firstly give scatterplots between selected measures of the respective track. The Spearman correlation coefficient is reported instead of the Pearson correlation coefficient. This is because we are only interested whether there is a correlation between the rankings of complexities, regardless of whether this is a linear relationship. We therefore also use the local regression smoothers in the plots (loess) rather than linear models (lm). Note: warning messages are disabled here as there are datasets with NAs, and for each plot this throws a warning message using the ggpairs() plotting function. NAs are dealt with by removing the entire row containing an NA value.

```
track.a.scatterplot <- ggpairs(track.a.short,
                        lower = list(continuous = wrap("smooth_loess", alpha = 0.3,
                                                       lwd = 0.5, size = 2)),
                        upper = list(continuous = wrap('cor', method = "spearman"))) +
                        theme_bw() +
                        theme(axis.text.x = element_text(angle = 90, hjust = 1))
print(track.a.scatterplot)
```

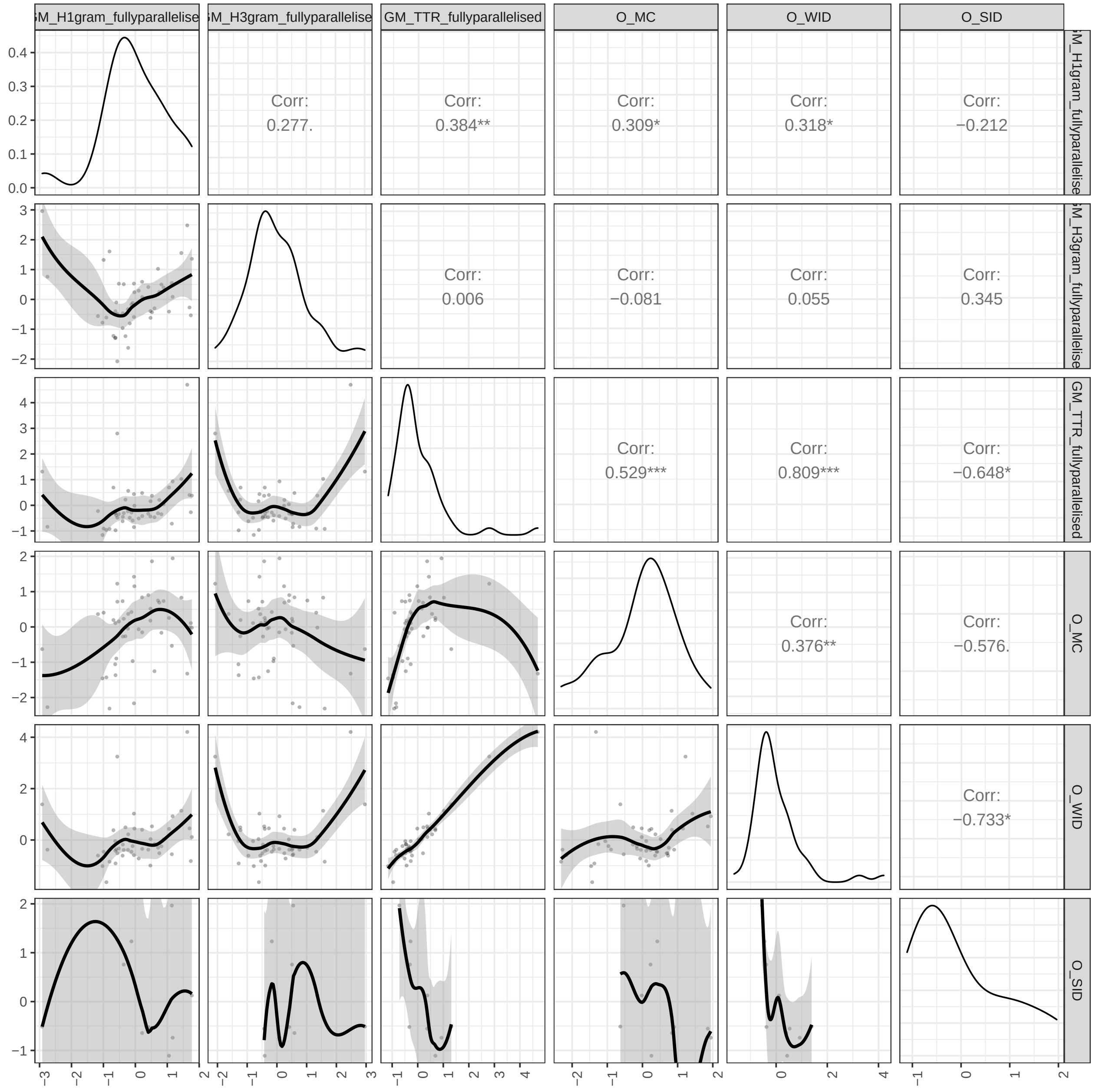

TRACK B

Same for the Track B data. Not all measures are included here (there would be 24). To include them all, the "columns" argument in the code below might be removed.

```
track.b.scatterplot <- ggpairs(track.b.short,
                           lower = list(continuous = wrap("smooth_loess", alpha = 0.3,
                                                          lwd = 0.5, size = 2)),
                           upper = list(continuous = wrap('cor', method = "spearman")),
                           columns = c("BV_n_tokens", "BV_char_per_tok", "BV_avg_links_len",
                                       "CR_inflection_accuracy", "CR_ttr",
                                 "S_idMean", "S_idSD", "SI_dm", "SI_hm", "SI_dep_dl",
                                 "SBS_INF", "SBS_DER")) +
```

```
                            theme_bw() +
                            theme(axis.text.x = element_text(angle = 90, hjust = 1))
print(track.b.scatterplot)
```

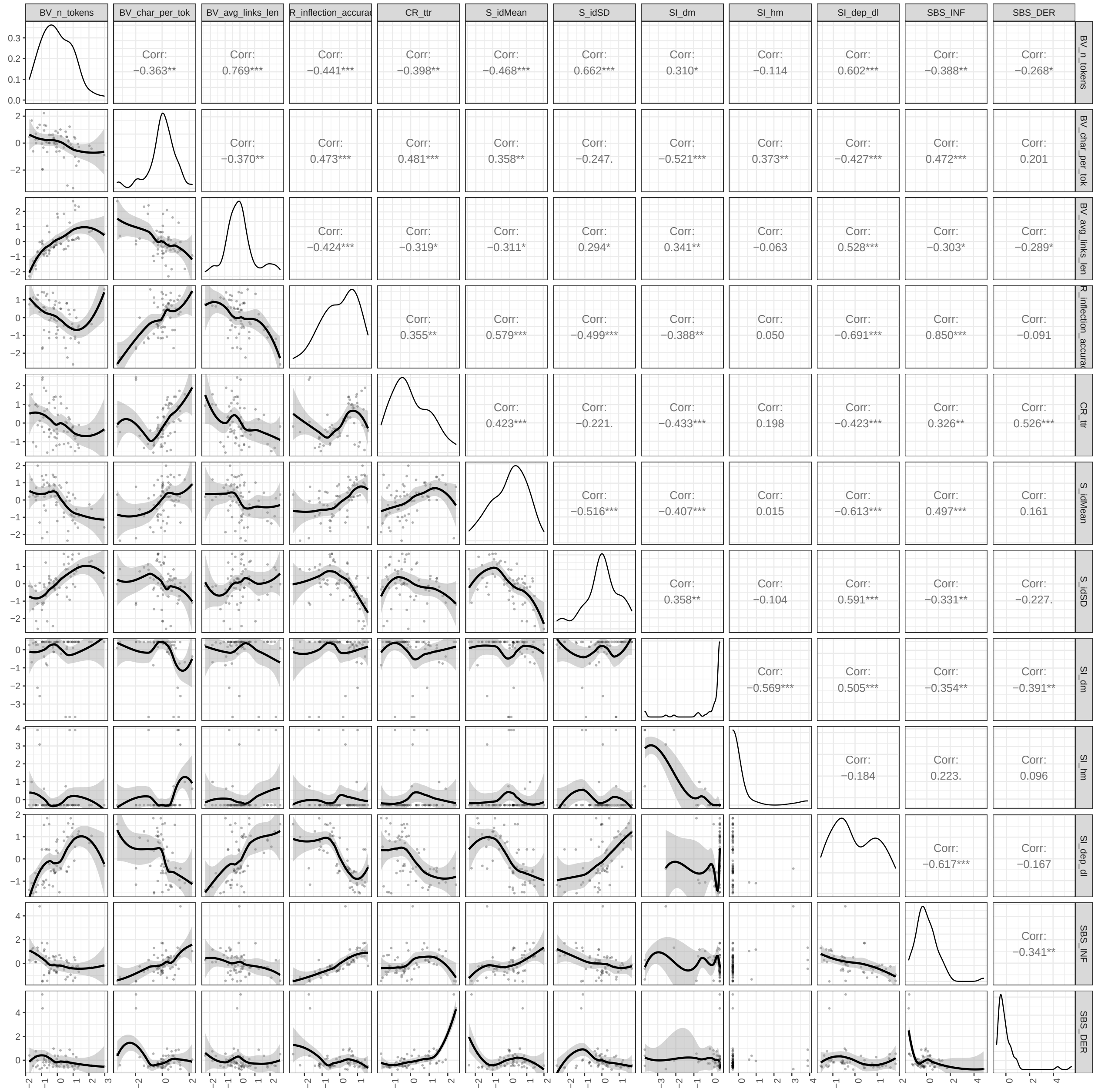

Significant Correlations after Correction for Multiple Testing

Not all of the correlations displayed above are going to be significant after correcting for multiple testing. We therefore use the corr.test() function here, since it enables us to choose a correction method, i.e. Holm-Bonferroni. The Bonferroni method would be more conservative, however, it is pointed out in MacDonald (2014, p. 254-260) that it is appropriate only when tests are independent of one another. Since we here run pairwise tests by complexity measures, our tests are not independent (the same measure is tested against others multiple times). We therefore apply the Holm-Bonferroni method (see also the descriptions in the

vignette invoked by the command "?p.adjust()"). Note that NAs are here deleted in pairs of columns, rather than across a whole row.

TRACK A

Calculate Spearman rank correlations with p-values adjusted by the Holm-Bonferroni method.

```
cor.results.a <- corr.test(track.a.short, method = "spearman",
                           use = "pairwise.complete.obs", adjust = "holm")
```

Give correlogram of selected measures. Note that the type has to be "upper" here, since the corrected p-values are in the upper triangle of the matrix, while the unadjusted p-values are in the lower triangle (thanks to Sonia Petrini for pointing this out).

```
correlogram.TrackA <- ggcorrplot(cor.results.a$r, p.mat = cor.results.a$p, type = "upper",
     outline.col = "white", colors = c("#3C77AE", "white", "#AE3C3C"),
     lab = T, insig = "pch")
correlogram.TrackA
```

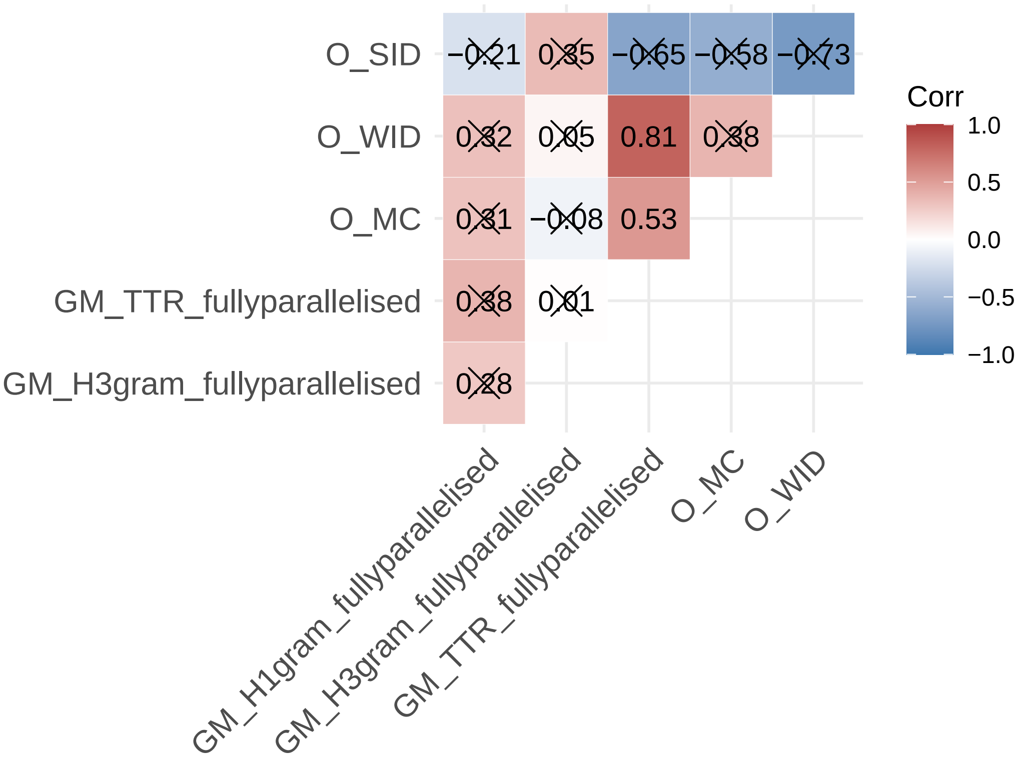

Safe to file.

```
ggsave("Figures/Corrs/correlogram_TrackA.pdf", correlogram.TrackA,
       dpi = 300, scale = 1, width = 5.5, height = 5.5, device = cairo_pdf)
```

TRACK B

Since Indo-European languages are over-represented in Track B (Universal Dependencies data), we here first give an overall correlational analysis regardless of language family, and then we give separate analyses for Indo-European and non-Indo-European languages. This helps to get an idea to what extent the overall correlational patterns are driven by Indo-European languages.

Overall Analyses

Reorder columns to have measures of the same domain together.

```
col_order <- c("BV_char_per_tok", "CR_inflection_accuracy", "CR_ttr",
               "CR_msp", "CR_mfe", "CR_cfe_form_feat", "CR_cfe_feat_form",
               "SI_dm", "SI_hm", "SBS_INF", "SBS_DER", "BV_n_tokens",
               "BV_verbal_head_per_sent", "BV_verbal_root_perc", "BV_avg_links_len",
               "BV_avg_subordinate_chain_len", "BV_subordinate_pre",
               "BV_subordinate_post", "BV_avg_verb_edges", "S_idMean", "S_idSD", "SI_dep_dl")
track.b.short.reorder <- track.b.short[, col_order]
#track.b.short.reorder
```

Same as above for Track A.

```
cor.results.b <- corr.test(track.b.short.reorder, method = "spearman",
                          use = "pairwise.complete.obs", adjust = "holm")
# produce correlogram
correlogram.TrackB <- ggcorrplot(cor.results.b$r, p.mat = cor.results.b$p, type = "upper",
     outline.col = "white", colors = c("#3C77AE", "white", "#AE3C3C"),
     lab = T, insig = "pch") +
  geom_segment(aes(x = 11.5, y = 10.5, xend = 11.5, yend = 21.5)) +
  geom_segment(aes(x = 0.5, y = 10.5, xend = 11.5, yend = 10.5))
correlogram.TrackB
```

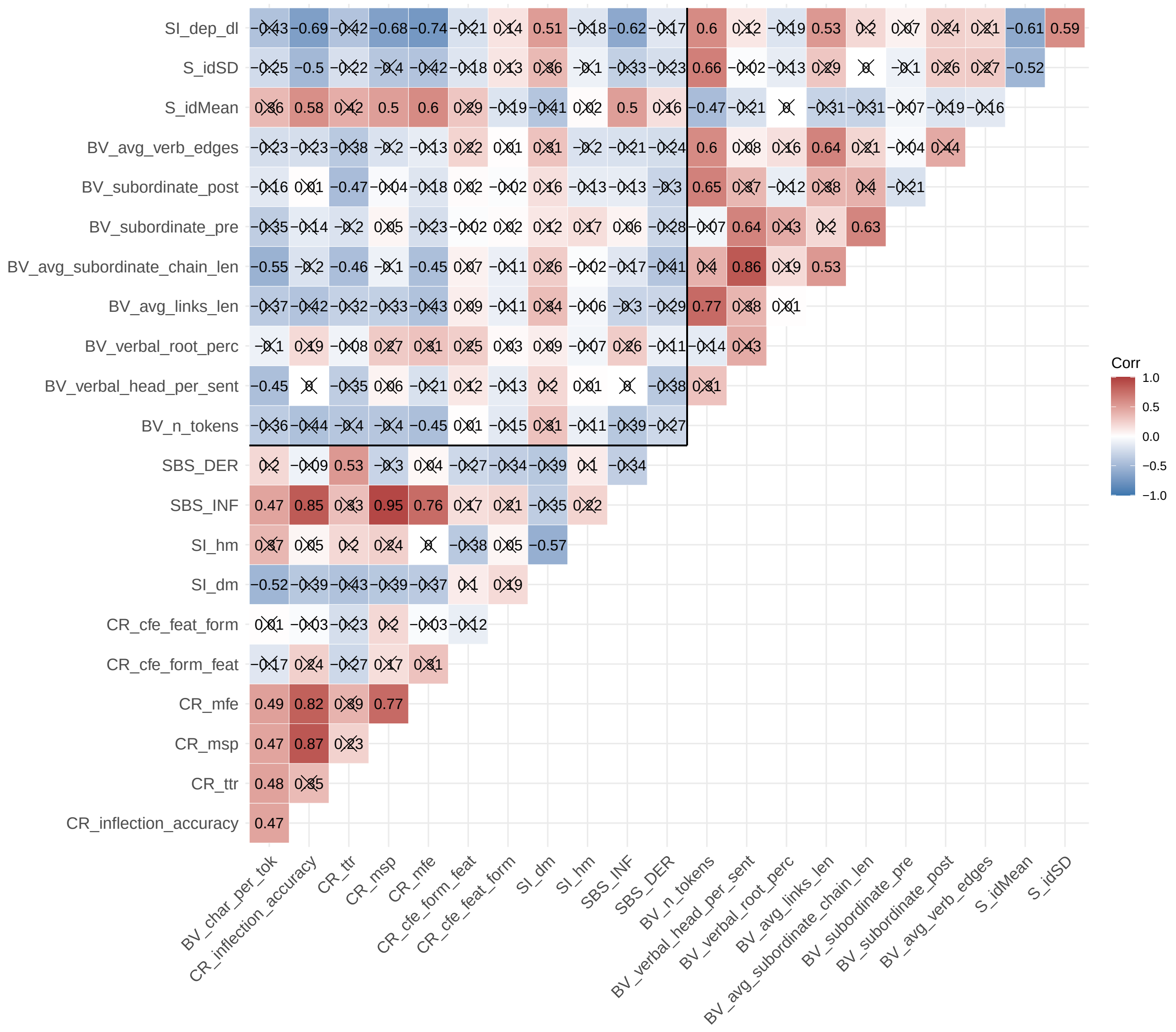

Safe to file.

```
ggsave("Figures/Corrs/correlogram_TrackB.pdf", correlogram.TrackB,
       dpi = 300, scale = 1, width = 12, height = 12, device = cairo_pdf)
```

Analyses for Indo-European languages only

Reorder columns to have measures of the same domain together.

```
col_order <- c("BV_char_per_tok", "CR_inflection_accuracy", "CR_ttr",
               "CR_msp", "CR_mfe", "CR_cfe_form_feat", "CR_cfe_feat_form",
               "SI_dm", "SI_hm", "SBS_INF", "SBS_DER", "BV_n_tokens",
               "BV_verbal_head_per_sent", "BV_verbal_root_perc", "BV_avg_links_len",
               "BV_avg_subordinate_chain_len", "BV_subordinate_pre",
               "BV_subordinate_post", "BV_avg_verb_edges", "S_idMean", "S_idSD", "SI_dep_dl")
track.b.short.IE.reorder <- track.b.IE.short[, col_order]
```

Get correlation results.

```r
cor.results.b.IE <- corr.test(track.b.short.IE.reorder, method = "spearman",
                         use = "pairwise.complete.obs", adjust = "holm")
```

```
## Warning in cor(x, use = use, method = method): the standard deviation is zero
```

```r
# produce correlogram
correlogram.TrackB.IE <- ggcorrplot(cor.results.b.IE$r, p.mat = cor.results.b.IE$p,
     type = "upper", outline.col = "white", colors = c("#3C77AE", "white", "#AE3C3C"),
     lab = T, insig = "pch", title = "Indo-European languages") +
  geom_segment(aes(x = 11.5, y = 10.5, xend = 11.5, yend = 21.5)) +
  geom_segment(aes(x = 0.5, y = 10.5, xend = 11.5, yend = 10.5))
#correlogram.TrackB.IE
```

Analyses for non-Indo-European languages only

Reorder columns to have measures of the same domain together.

```r
col_order <- c("BV_char_per_tok", "CR_inflection_accuracy", "CR_ttr",
               "CR_msp", "CR_mfe", "CR_cfe_form_feat", "CR_cfe_feat_form",
               "SI_dm", "SI_hm", "SBS_INF", "SBS_DER", "BV_n_tokens",
               "BV_verbal_head_per_sent", "BV_verbal_root_perc", "BV_avg_links_len",
               "BV_avg_subordinate_chain_len", "BV_subordinate_pre",
               "BV_subordinate_post", "BV_avg_verb_edges", "S_idMean", "S_idSD", "SI_dep_dl")
track.b.short.nonIE.reorder <- track.b.nonIE.short[, col_order]
```

Get correlation results.

```r
cor.results.b.nonIE <- corr.test(track.b.short.nonIE.reorder, method = "spearman",
                         use = "pairwise.complete.obs", adjust = "holm")
# produce correlogram
correlogram.TrackB.nonIE <- ggcorrplot(cor.results.b.nonIE$r, p.mat = cor.results.b.nonIE$p,
     type = "upper", outline.col = "white", colors = c("#3C77AE", "white", "#AE3C3C"),
     lab = T, insig = "pch", title = "Non-Indo-European languages") +
  geom_segment(aes(x = 11.5, y = 10.5, xend = 11.5, yend = 21.5)) +
  geom_segment(aes(x = 0.5, y = 10.5, xend = 11.5, yend = 10.5))
#correlogram.TrackB.nonIE
```

Combine plots in one panel.

```r
grid.arrange(correlogram.TrackB.IE, correlogram.TrackB.nonIE, ncol = 2)
```

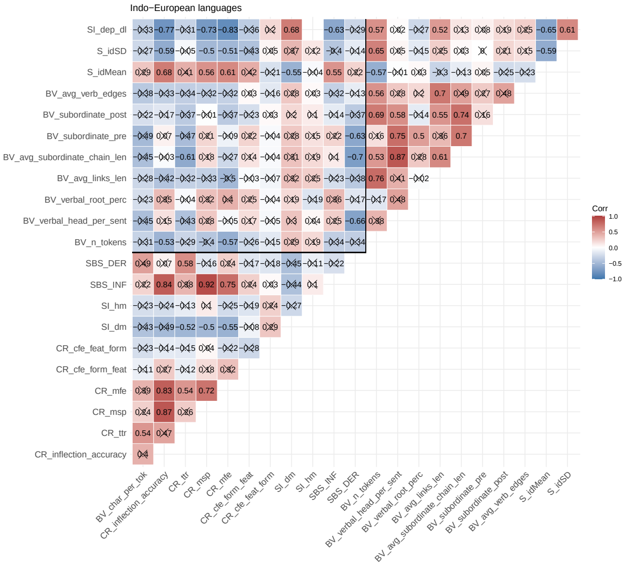

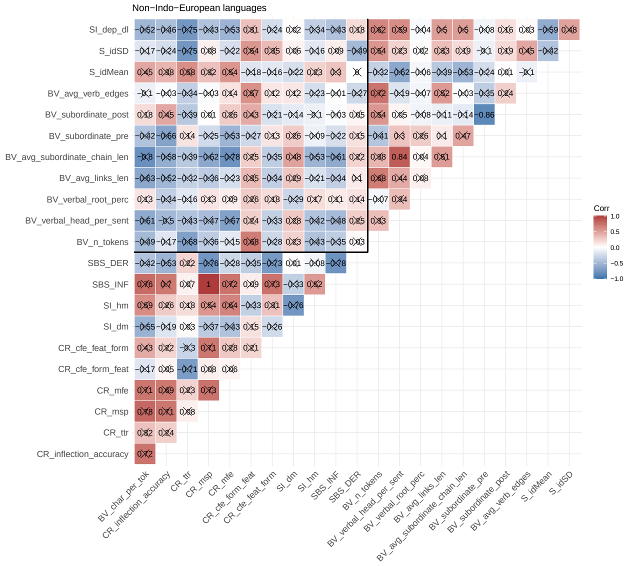

Interpretation: The correlograms for Indo-European languages and non-Indo-European (Japanese, Mandarin Chinese, Arabic, etc.) look similar in the sense that in both we have mainly positive correlations between measures in the same domain (i.e. red colors in the lower left and upper right triangle), and mainly negative correlations between the domains (i.e. blue colors) in the upper left square. One difference between the correlograms is that there are more significant correlations for the IE languages. This is expected since there are considerably more IE languages in Track B (i.e. 31) than non-IE languages (i.e. 13), which gives us more statistical power to detect significant correlations.

Detailed Scatterplots

We here plot the highest *positive* and *negative* correlations (in terms of Spearman coefficients) which are found between measures proposed by *different participants* (there are many measures by the same participants that highly correlate). These are hand-picked from the correlograms above.

TRACK A (Highest Positive Correlation)

```
track.a.positive.detailed <- ggplot(track.a, aes(x = GM_TTR_fullyparallelised, y = O_WID)) +
  geom_point(alpha = 0.3) +
  geom_smooth(method = loess, alpha = 0.3) +
  geom_label_repel(data = track.a[track.a$language == "Fijian"
                                  | track.a$language == "Sango"
                                  | track.a$language == "Vietnamese"
                                  | track.a$language == "English"
                                  | track.a$language == "Georgian"
                                  | track.a$language == "Russian"
                                  | track.a$language == "Swahili"
                                  | track.a$language == "Basque"
                                  | track.a$language == "Finnish"
                                  | track.a$language == "Turkish"
                                  | track.a$language == "Korean"
                                  | track.a$language == "Kalaallisut"
                                  | track.a$language == "Burmese", ],
```

```
                   min.segment.length = 0,
                   #nudge_x = 0.1,
                   aes(label = language),
                       size = 3) +
  ggtitle("a) High Positive Correlation Track A (r = 0.81)") +
  xlab("Type-Token-Ratio (GM_TTR_fullyparallelized)") +
  ylab("Word Information Density (O_WID)") +
  theme(legend.position = "none")
# track.a.positive.detailed
```

Some comments: This plot shows that the Type-Token Ratio (TTR) and the Word Information Density (WID) are highly positively correlated across the languages of the Parallel Bible Corpus sample. Burmese (mya) is an outlier here with very high TTR and WID. This is an artifact of the writing system, since it does not delimit orthographic words by white spaces, but rather phrases. For Kalaallisut, on the other hand, the result makes sense also in the light of morphological typology (if we accept the latinized writing proposed for this language). Some of the low TTR languages include Sango (sag), Fijian (fij), Thai (tha), and Yoruba (yor).

TRACK B (Highest Positive Correlation)

```
#track.b <- track.b[track.b$id != "uig", ] # remove the outlier Uyghur (uig)

track.b.positive.detailed <- ggplot(track.b, aes(x = CR_msp, y = SBS_INF)) +
  geom_point(alpha = 0.3) +
  geom_smooth(method = loess, alpha = 0.3) +
  geom_label_repel(data = track.b[track.b$language == "Chinese"
                                  | track.b$language == "Vietnamese"
                                  | track.b$language == "English"
                                  | track.b$language == "Russian"
                                  | track.b$language == "Old Church Slavonic"
                                  | track.b$language == "Basque"
                                  | track.b$language == "Finnish"
                                  | track.b$language == "Turkish"
                                  | track.b$language == "Latin"
                                  | track.b$language == "Uyghur"
                                  | track.b$language == "Ancient Greek", ],
                   min.segment.length = 0,
                   #nudge_x = 0.1,
                   aes(label = language),
                   size = 3) +
  ggtitle("b) High Positive Correlation Track B (r = 0.95)") +
  xlab("Mean Size of Morphological Paradigms (CR_msp)") +
  ylab("Inflectional Entropy (SBS_INF)") +
  theme(legend.position = "none")
# track.b.positive.detailed
```

Some comments: This plot shows the correlation between the so-called Mean Size of Morphological Paradigms (MSP), which is defined by CR as “simply the number of word-form types divided by the number of lemma types”, and the difference in unigram entropy of word tokens in the original texts and the lemmatized texts (INF) as defined by SBS. It is certainly not unexpected, but reassuring, to see these measures highly correlated. The outlier to the high end Uyghur (uig) is likely *not* an artifact, as this language indeed has many productive morphological paradigms. Other languages to the high end of morphological complexity include Ancient

Greek (grc), Classical Latin (lat), Turkish (tur), and Old Church Slavonic (chu). Languages to the low end are Vietnamese (vie), Indonesian (ind), Mandarin Chinese (cmn), and Afrikaans (afr). Note that the very low morphological complexity scores of Korean (kor) are an artifact of the way the Korean data is presented in the UD. Namely, the "lemmas" given for Korean are actually merely morphologically segmented forms rather than inflectionally neutralized forms as for the other languages. Thus, it makes sense that the MSP is exactly 1 and the INF is 0 for Korean.

TRACK A (Highest Negative Correlation)

```
track.a.negative.detailed <- ggplot(track.a, aes(x = GM_TTR_fullyparallelised, y = O_SID)) +
  geom_point(alpha = 0.3) +
  geom_smooth(method = loess, alpha = 0.3) +
  geom_label_repel(data = track.a,
                   min.segment.length = 0,
                   #nudge_x = 0.1,
                   aes(label = language),
                       size = 3) +
  ggtitle("c) High Negative Correlation Track A (r = -0.65)") +
  xlab("Type-Token-Ratio (GM_TTR_fullyparallelized)") +
  ylab("Syllable Information Density (O_SID)") +
  theme(legend.position = "none") +
  theme(legend.position = "none") +
  xlim(0.05, 0.4)
# track.a.negative.detailed
```

Some comments: This plot shows a negative correlation between type-token-ratios in parallel texts (GM_TTR_fullyparallelised) and syllable information density (O_SID). This can be seen as a trade-off between the diversity of word types and the information carried by syllables. Languages with agglutinative morphology, e.g. Finnish and Turkish have many word types, but low syllable information density. Languages with rather isolating morphology, e.g. English and French, have fewer word types, but more information-dense syllables. Note that this correlation is not significant after Holm-Bonferroni correction. Arguably, this has to do with the low number of languages for which syllabification is available. This leaves us with only 9 data points.

Track B (Highest Negative Correlation)

```
track.b <- track.b[track.b$language != "Korean", ] # remove the outlier Korean

track.b.negative.detailed <- ggplot(track.b, aes(x = CR_mfe, y = SI_dep_dl)) +
  geom_point(alpha = 0.3) +
  geom_smooth(method = loess, alpha = 0.3) +
  geom_label_repel(data = track.b[track.b$language == "Chinese"
                                  | track.b$language == "Vietnamese"
                                  | track.b$language == "German"
                                  | track.b$language == "English"
                                  | track.b$language == "Hungarian"
                                  | track.b$language == "Greek"
                                  | track.b$language == "Russian"
                                  | track.b$language == "Old Church Slavonic"
                                  | track.b$language == "Basque"
```

```
                                  | track.b$language == "Finnish"
                                  | track.b$language == "Turkish"
                                  | track.b$language == "Latin"
                                  | track.b$language == "Uyghur"
                                  | track.b$language == "Ancient Greek", ],
                    min.segment.length = 0,
                   #nudge_x = 0.1,
                   aes(label = language),
                   size = 3) +
  ggtitle("d) High Negative Correlation Track B (r = -0.74)") +
  xlab("Morphological Feature Entropy (CR_mfe)") +
  ylab("Dependency Length in Possessive NP (SI_dep_dl)") +
  theme(legend.position = "none")
# track.b.negative.detailed
```

Combine Scatterplots

We here combine the four scatterplots with some of the highest positive and negative correlations in one panel.

```
scatterplots <- grid.arrange(track.a.positive.detailed, track.b.positive.detailed,
                             track.a.negative.detailed, track.b.negative.detailed, nrow = 2)
```

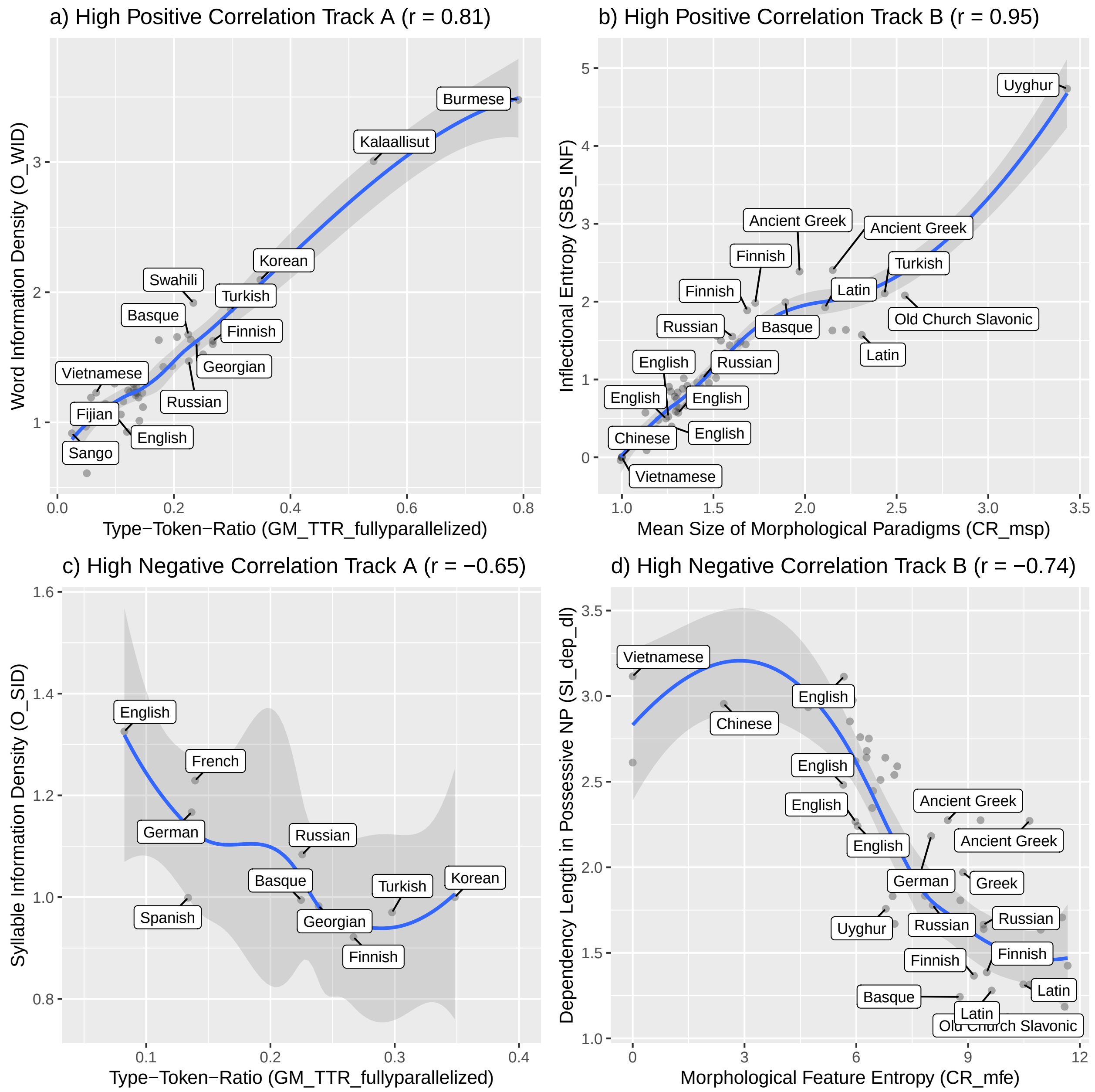

Safe to file.

```
ggsave("Figures/Corrs/scatterplots.pdf", scatterplots, dpi = 300,
       scale = 1, width = 9, height = 9, device = cairo_pdf)
```

Conclusions

Some more general observations based on these analyses include:

- Many of the measures proposed by the same participants have high positive correlations. This is the case, for instance, for the measures proposed by GM in Track A, but also measures of BV in Track B. In the case of GM, this is because many of the measures are virtually the same, but with minor shades of modification. In the case of BV, while at first sight the measures seem to conceptually differ, they

essentially boil down to the same underlying causes. For example, the number of tokens in a sentence highly predicts the average maximal depth of a tree over the sentence. So, arguably, most of these positive intra-participant correlations are driven by redundancy in the proposed measures.

- There are several strong positive correlations between simple measures relating to the number of types and tokens (GM_TTR_fullyparallelised, BV_n_tokens, etc.), and measures of information density (O_WID, S_idSD). Interestingly, this is the case for both tracks, since Oh used the Bible texts, and Semenuks used the UD. Information density is generally assumed to be a measure which has psycholinguistic relevance in terms of language processing. The fact that it is highly predictable by some of the simplest word frequency measures (TTR) potentially goes to show that the underlying principles driving complexity are similar across different conceptualizations and measures.
- An interesting negative correlation in Track A is found between syllable information density (O_SID) and measures of lexical diversity like TTR (although there are few data points in O_SID, and the correlation is not significant after correction for multiple testing). This is potential evidence for a trade-off between syllable complexity and word complexity reported also in earlier studies. A negative correlation in Track B which is robust after correction, and potentially interesting, is that the dependency lengths in noun phrases with marked possessives (SI_dep_dl) apparently are in an inverse relationship with different measures of inflectional complexity.

References

McDonald, J.H. (2014). Handbook of Biological Statistics (3rd ed.). Sparky House Publishing, Baltimore, Maryland. online at http://www.biostathandbook.com
