# Supplementary material for: Complexity trade-offs and equi-complexity in natural languages: a meta-analysis
Source: Linguist Vanguard. 2022 Oct 14;9(Suppl1):9–25. doi: 10.1515/lingvan-2021-0054 (PMC10234276; doi:10.1515/lingvan-2021-0054)
Supplement: Supplementary file 5 — Supplementary Material Details [file j_lingvan-2021-0054_suppl_005.pdf]

Appendix 5: Equi-Complexity Analyses

Chris Bentz

July 20, 2022

Session Info

Give the session info (reduced).

```
## [1] "R version 3.6.3 (2020-02-29)"
```

```
## [1] "x86_64-pc-linux-gnu"
```

Load Packages

Load packages. If they are not installed yet on your local machine, use install.packages() to install them.

```
library(readr)
library(MASS)
library(tidyr)
library(ggplot2)
library(ggridges)
library(scales)
library(plyr)
library(rstatix)
```

Give the package versions.

```
##  rstatix     plyr   scales ggridges  ggplot2    tidyr     MASS    readr
##  "0.7.0"  "1.8.6"  "1.1.1"  "0.5.3"  "3.3.5"  "1.1.4" "7.3-54"  "2.0.2"
```

Introduction

This file gives the R code to replicate our equi-complexity analyses for the empirical data gathered from 44 languages. Note that we here use languages of Track B, since it gives several measures of both domains (morphology and syntax).

Load Data

Load results of all participants in the UD track directly from github repo by using the "readr" library. We here only use Track B as we have measures from different domains (morphology, syntax) in this track, while in Track A we have mainly morphological complexity measures.

```
#TRACK B (Universal Dependencies, UD)
brunato.results  <- read_csv("https://raw.githubusercontent.com/IWMLC/language-complexity-metrics/maste
coltekin.results  <- read_csv("https://raw.githubusercontent.com/IWMLC/language-complexity-metrics/mast
semenuks.results  <- read_csv("https://raw.githubusercontent.com/IWMLC/language-complexity-metrics/mast
sinnemaki.results  <- read_csv("https://raw.githubusercontent.com/IWMLC/language-complexity-metrics/mas
sozinova.results  <- read_csv("https://raw.githubusercontent.com/IWMLC/language-complexity-metrics/mast
```

Load file with meta-information on the measures. This is slightly adapted from a file prepared by Katharina Ehret and Alice Blumenthal-Dramé in the context of the Interactive Workshop on Measuring Language Complexity (IWMLC 2019).

```
meta.info  <- read_csv("https://raw.githubusercontent.com/IWMLC/language-complexity-metrics/master/meas
```

Data Pre-Processing

Create a single data frame merging results together using the Reduce() and merge() functions of base R.

```
# by default the merge() function uses columns with the same names
# to merge on, i.e. "id" and "language" in our case.
results <- Reduce(merge, list(brunato.results, coltekin.results, semenuks.results,
                              sinnemaki.results, sozinova.results))
```

Give some simple statistics for this data frame of results.

```
nrow(results) # i.e. number of different UD corpora
```

```
## [1] 63
```

```
length(unique(results$language)) # i.e. number of different languages
```

```
## [1] 44
```

```
unique(results$language) # output the language names
```

```
##  [1] "Afrikaans"          "Arabic"              "Bulgarian"
##  [4] "Catalan"            "Czech"               "Old Church Slavonic"
##  [7] "Chinese"            "Danish"              "German"
## [10] "Estonian"           "Greek"               "English"
## [13] "Basque"             "Persian"             "Finnish"
## [16] "French"             "Gothic"              "Ancient Greek"
## [19] "Hebrew"             "Hindi"               "Croatian"
## [22] "Hungarian"          "Indonesian"          "Italian"
## [25] "Japanese"           "Korean"              "Latin"
## [28] "Latvian"            "Dutch"               "Norwegian"
## [31] "Polish"             "Portuguese"          "Romanian"
## [34] "Russian"            "Slovak"              "Slovenian"
## [37] "Spanish"            "Serbian"             "Swedish"
## [40] "Turkish"            "Uyghur"              "Ukrainian"
## [43] "Urdu"               "Vietnamese"
```

```
ncol(results)-2 # i.e. number of complexity measures
```

```
## [1] 27
```

Invert the values (by subtracting them from 1) for the measure "CR_inflection_accuracy". Note that higher values in the original measure mean *lower* rather than higher complexity.

```r
results$CR_inflection_accuracy <- 1-results$CR_inflection_accuracy
```

Center and scale all numerical columns to make them more commensurable.

```r
# scale and add meta-information columns again
results.scaled <- cbind(results[1:2], scale(results[3:29]))
```

Remove certain measures. To include all measures, this code can just be commented out. In Track B, some measures given by the same team have strong positive correlations, e.g. the number of tokens in a sentence (BV_n_tokens) and the average number of tokens per clause (BV_avg_token_per_clause). We hence just keep one of the strongly correlated measures to not inflate the number of strongly correlated data points. Also there are measures with many NAs in Track B, i.e. "SI_double_dl", "SI_head_dl", "SI_zero_dl", which are removed here.

```r
# Remove measures in Track B
results.scaled <- results.scaled[ , -which(names(results.scaled) %in%
                                            c("BV_avg_max_depth","BV_avg_token_per_clause",
                                              "SI_double_dl", "SI_head_dl", "SI_zero_dl"))]
nrow(results.scaled)
```

```
## [1] 63
```

Transform data frames from wide format to long format (this is necessary for later plotting and analyses by columns rather than rows).

```r
results.long <- gather(results.scaled, key = measure, value = value, BV_n_tokens:SBS_DER)
```

Remove rows which contain NAs.

```r
results.long <- results.long[complete.cases(results.long), ]
nrow(results.long)
```

```
## [1] 1380
```

Add meta-information on complexity measures.

```r
results.long <- merge(results.long, meta.info[, 2:4], by = "measure")
```

Generate data frames for subdomains (morphology, syntax). This can be used for comparison with aggregated results.

```r
results.morph <- results.long[results.long$domain == "morphology", ]
results.syntax <- results.long[results.long$domain == "syntax", ]
```

We might have a closer look also at languages for which there are several treebanks (here we choose English, since it has most treebanks, i.e. four) in order to check whether the statistical results are similar for different treebanks of the same language (this is checked for the case of effect sizes further below).

```r
# select languages with several treebanks
selection <- c("English")
results.long.selected <- results.long[results.long$language %in% selection, ]
```

Density Distributions

Plot density distributions of complexity measurements by language and treebank. Individual measurements for each complexity measure (and different treebanks if available) are plotted as dots. The central value (0) is indicated by a vertical gray line for visual reference.

Create data frame with both domains + "overall", this is necessary for the plots below.

```
results.overall <- results.long
results.overall$domain <- rep("overall", times = nrow(results.long))
results.plot <- rbind(results.overall, results.long)
```

By Language

Plot density distributions by language with indication of median values. Note that we limit the plotting range on the x-axis to -5 to 5 (standard deviations), meaning that extreme outliers are not plotted. There are 4 outliers dropped here, which results in a warning message "Removed 4 rows containing non-finite values (stat_density_ridges)".

```
density.plot <- ggplot(results.plot, aes(x = value, y = domain, fill = domain)) +
  geom_vline(aes(xintercept = 0), color = "darkgrey") +
  stat_density_ridges(quantile_lines = TRUE, quantiles = 0.5,
                      rel_min_height = 0.01, scale = 0.6, alpha = 0.5,
                      jittered_points = TRUE, point_size = 0.5,
                      size = 0.25, position = position_points_jitter(height = 0.1)) +
  facet_wrap(~ language, ncol = 5) +
  xlim(-5, 5) +
  labs(x = "Complexity Value", y = "Density") +
  theme_bw() +
  theme(legend.position = "none")
print(density.plot)
```

```
## Warning: Removed 4 rows containing non-finite values (stat_density_ridges).
```

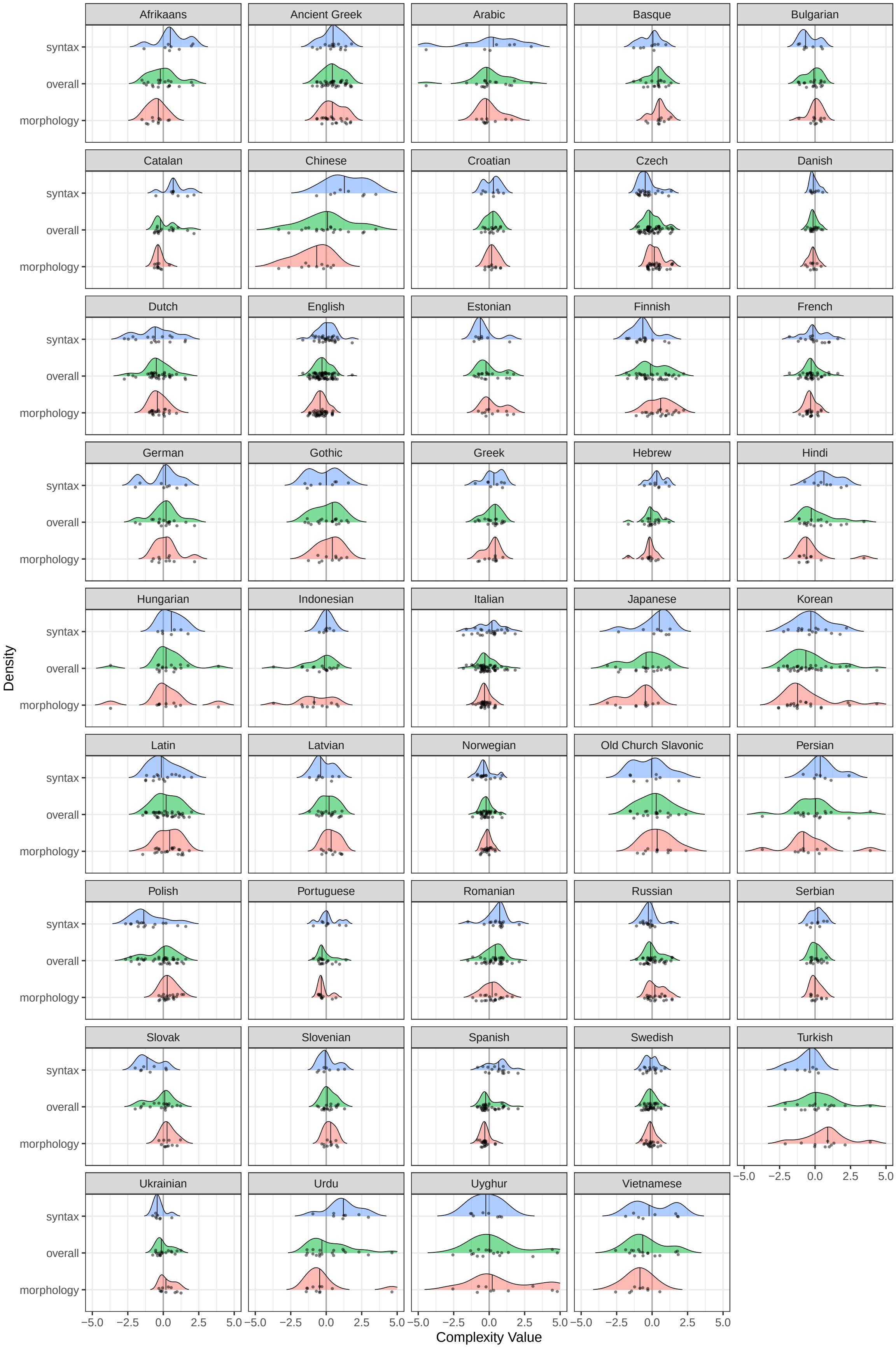
Afrikaans
Ancient Greek
Arabic
Basque
Bulgarian
Catalan
Chinese
Croatian
Czech
Danish
Dutch
English
Estonian
Finnish
French
German
Gothic
Greek
Hebrew
Hindi
Hungarian
Indonesian
Italian
Japanese
Korean
Latin
Latvian
Norwegian
Old Church Slavonic
Persian
Polish
Portuguese
Romanian
Russian
Serbian
Slovak
Slovenian
Spanish
Swedish
Turkish
Ukrainian
Urdu
Uyghur
Vietnamese
syntax
overall
morphology
Density
Complexity Value
−5.0
−2.5
0.0
2.5
5.0

Save figure to file.

```
ggsave("Figures/EquiCompl/density_plot.pdf", density.plot,
       dpi = 300, scale = 1, width = 10, height = 15, device = cairo_pdf)
```

By Treebank

For visual comparison, also plot the density distributions faceted by treebank rather than by language.

```
density.plot.treebanks <- ggplot(results.plot, aes(x = value, y = domain, fill = domain)) +
  geom_vline(aes(xintercept = 0), color = "darkgrey") +
  stat_density_ridges(quantile_lines = TRUE, quantiles = 0.5,
                      rel_min_height = 0.01, scale = 0.6, alpha = 0.5,
                      jittered_points = TRUE, point_size = 0.5,
                      size = 0.25, position = position_points_jitter(height = 0.1)) +
  facet_wrap(~ id, ncol = 5) +
  xlim(-5, 5) +
  labs(x = "Complexity Value", y = "Density") +
  theme_bw() +
  theme(legend.position = "none")
print(density.plot.treebanks)
```

```
## Warning: Removed 4 rows containing non-finite values (stat_density_ridges).
```

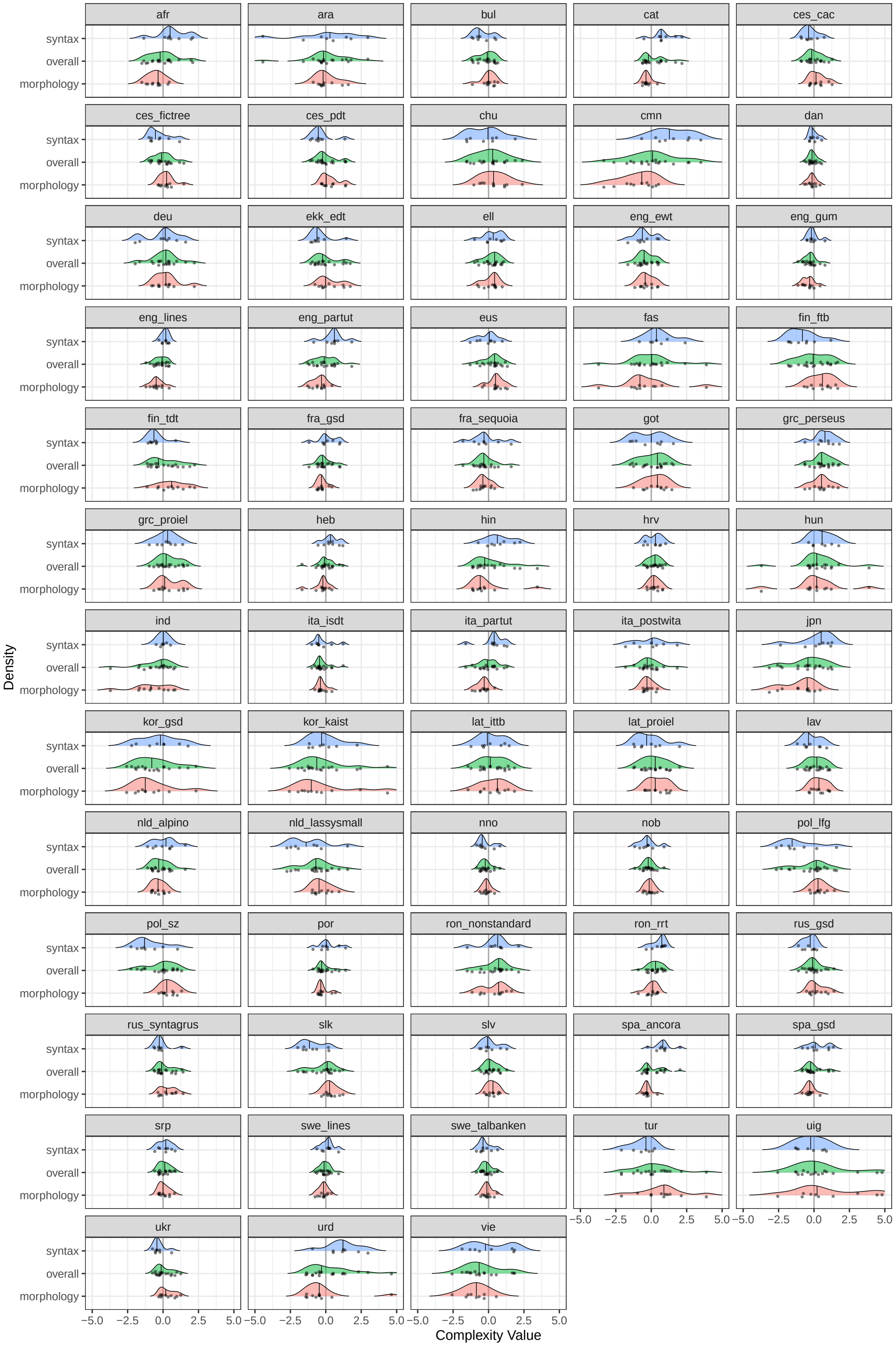

afr
ara
bul
cat
ces_cac
ces_fictree
ces_pdt
chu
cmn
dan
deu
ekk_edt
ell
eng_ewt
eng_gum
eng_lines
eng_partut
eus
fas
fin_ftb
fin_tdt
fra_gsd
fra_sequoia
got
grc_perseus
grc_proiel
heb
hin
hrv
hun
ind
ita_isdt
ita_partut
ita_postwita
jpn
kor_gsd
kor_kaist
lat_ittb
lat_proiel
lav
nld_alpino
nld_lassysmall
nno
nob
pol_lfg
pol_sz
por
ron_nonstandard
ron_rrt
rus_gsd
rus_syntagrus
slk
slv
spa_ancora
spa_gsd
srp
swe_lines
swe_talbanken
tur
uig
ukr
urd
vie
syntax
overall
morphology
Density
−5.0 −2.5 0.0 2.5 5.0
Complexity Value

Statistical Tests

Run statistical tests to assess whether the aggregate complexity distributions for certain pairs of languages differ significantly from one another. Note that for this sample of 44 languages there are 44*(44-1)/2 = 946 possible pairwise comparisons.

Overview of median values

Get mean, median, and standard deviation values.

```
# get median value for each language
med <- ddply(results.long, "language", summarise, grp.median = median(value))
# get mean value for each language
mu <- ddply(results.long, "language", summarise, grp.mean = mean(value))
# get standard deviation for each language
sdev <- ddply(results.long, "language", summarise, grp.sdev = sd(value))
```

Give an overview of languages with highest and lowest median complexity values.

```
stats.df <- cbind(mu, med[, 2], sdev[, 2])
colnames(stats.df) <- c("language", "mu", "med", "sdev")
stats.df.sorted <- stats.df[order(-stats.df$med),]
# round values to two decimal places,  the "-1" excludes column 1
stats.df.sorted[, -1] <- round(stats.df.sorted[, -1], 2)
print(stats.df.sorted)
```

```
##               language    mu   med sdev
## 2        Ancient Greek  0.49  0.43 0.74
## 17              Gothic  0.08  0.43 0.95
## 33            Romanian  0.34  0.43 0.74
## 18               Greek  0.22  0.43 0.60
## 4               Basque  0.25  0.43 0.69
## 29 Old Church Slavonic  0.15  0.29 1.11
## 8             Croatian  0.22  0.26 0.45
## 26               Latin  0.23  0.20 0.90
## 27             Latvian  0.13  0.20 0.65
## 16              German  0.08  0.20 0.99
## 21           Hungarian  0.33  0.20 1.46
## 35             Serbian  0.10  0.12 0.41
## 43              Uyghur  0.80  0.12 2.48
## 36              Slovak -0.21  0.10 0.90
## 7              Chinese  0.05  0.09 1.77
## 37           Slovenian  0.20  0.08 0.51
## 31              Polish -0.21  0.06 1.11
## 40             Turkish  0.21  0.05 1.46
## 30             Persian -0.03  0.03 1.61
## 5            Bulgarian -0.17 -0.09 0.59
## 19              Hebrew  0.00 -0.09 0.62
## 34             Russian  0.07 -0.11 0.63
## 14             Finnish  0.06 -0.12 1.05
## 22          Indonesian -0.46 -0.12 1.11
## 39             Swedish -0.10 -0.13 0.40
## 41           Ukrainian  0.04 -0.14 0.57
## 10              Danish -0.10 -0.14 0.31
```

```
## 3                Arabic  0.04 -0.16 1.53
## 9                 Czech  0.02 -0.16 0.66
## 6               Catalan  0.19 -0.20 0.81
## 1             Afrikaans -0.02 -0.20 1.02
## 28            Norwegian -0.19 -0.22 0.43
## 13             Estonian  0.00 -0.22 0.86
## 38              Spanish  0.05 -0.25 0.67
## 20                Hindi  0.18 -0.27 1.24
## 15               French -0.20 -0.28 0.64
## 32           Portuguese -0.07 -0.29 0.57
## 12              English -0.23 -0.29 0.59
## 23              Italian -0.17 -0.31 0.68
## 42                 Urdu  0.36 -0.31 1.62
## 24             Japanese -0.52 -0.43 1.30
## 11                Dutch -0.42 -0.49 0.93
## 25               Korean -0.26 -0.64 1.80
## 44           Vietnamese -0.45 -0.66 1.29
```

Output data frame as csv file.

```
write.csv(stats.df.sorted, file = "Tables/descriptiveStats.csv", row.names = F)
```

Normality

Check normality of data points by QQ-plot.

```
ggplot(results.long, aes(sample = value)) +
  stat_qq()
```

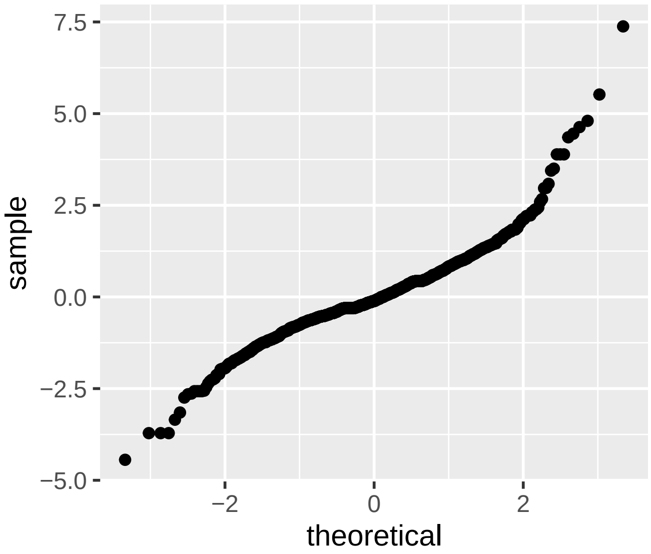

Choose statistical tests

Since there are some deviations from normality visible in the QQ-Plot above, we here choose a Wilcoxon test. If we supply two data vectors, then by default the command wilcox.test() runs a Wilcoxon rank sum test (for unpaired samples), and with "paired = T" a Wilcoxon signed rank test. Determining whether our data is "paired" or "unpaired" is tricky. On one hand, the same complexity measures were applied here across different languages (and corresponding text samples), and we show in our correlational analyses that the

values given by these measures often correlate. The samples of complexity measurements per language could thus be considered "related" in the general sense of Cahusac (2021, p. 56). On the other hand, however, there are still some NAs in the data, and, for some languages, e.g. Czech and English, there are several treebanks in the UD, meaning that there are data points which are not matched in the samples of other languages. Due to this structure of the data, we are forced to consider the vectors of complexity measurements "unpaired".

Note that pairwise.wilcox.test() is a function of the R-core stats package which runs multiple tests, i.e. for all groups in the "language" column in our case.

P-value adjustment for multiple comparisons: In case of multiple testing, we should account for the fact that the likelihood of finding a significant result by chance increases with the number of statistical tests. One of the most conservative methods to account for this is the so-called Bonferroni correction, i.e. multiplying the p-values with the number of tests. This method is appropriate when tests are independent of one another (MacDonald 2014, p. 254-260). Since we here run pairwise tests by languages, our tests are not independent (the same language is tested against others multiple times). We therefore apply the so-called Holm-Bonferroni method, which is less conservative. It does not assume independence between tests (see the descriptions in the vignette invoked by the command "?p.adjust()").

Tests for all measures (morphology and syntax)

We here run pairwise Wilcoxon tests and extract pairs of languages for which $p < 0.05$ (after Holm-Bonferroni correction). This is for all measures aggregated. Below we do the same for morphological and syntactic complexity measures separately.

```
# we add some random noise here to the value vector with
# the function jitter(), since we otherwise get warnings due to ties in the data
p.values <- pairwise.wilcox.test(jitter(results.long$value), results.long$language,
                      paired = F, p.adjust.method = "holm")
# print(p.values)
# get dataframe with pairs of languages where p < 0.05
m <- p.values$p.value
indices <- which(m < 0.05, arr.ind = T)
v1 <- rownames(m)[indices[, "row"]]
v2 <- colnames(m)[indices[, "col"]]
df <- cbind(v1, v2)
print(df)
```

```
##      v1          v2
## [1,] "Dutch"     "Ancient Greek"
## [2,] "English"   "Ancient Greek"
## [3,] "Italian"   "Ancient Greek"
## [4,] "Norwegian" "Ancient Greek"
## [5,] "Romanian"  "English"
```

Tests for morphological complexity measures

```
p.values.morph <- pairwise.wilcox.test(jitter(results.morph$value), results.morph$language,
                      paired = F, p.adjust.method = "holm")
# print(p.values.morph)
# we add some random noise here to the value vector with
# the function jitter(), since we otherwise get warnings due to ties in the data
# get dataframe with pairs of languages where p < 0.05
m <- p.values.morph$p.value
indices <- which(m < 0.05, arr.ind = T)
```

```
v1 <- rownames(m)[indices[, "row"]]
v2 <- colnames(m)[indices[, "col"]]
df <- cbind(v1, v2)
print(df)
```

```
##       v1         v2
##  [1,] "English"  "Ancient Greek"
##  [2,] "Italian"  "Ancient Greek"
##  [3,] "Spanish"  "Ancient Greek"
##  [4,] "English"  "Basque"
##  [5,] "Italian"  "Basque"
##  [6,] "Polish"   "Catalan"
##  [7,] "English"  "Czech"
##  [8,] "French"   "Czech"
##  [9,] "Italian"  "Czech"
## [10,] "Japanese" "Czech"
## [11,] "Spanish"  "Czech"
## [12,] "Finnish"  "English"
## [13,] "Polish"   "English"
## [14,] "Russian"  "English"
## [15,] "Polish"   "French"
## [16,] "Polish"   "Italian"
## [17,] "Polish"   "Japanese"
## [18,] "Spanish"  "Polish"
```

Tests for syntactic complexity measures

```
p.values.syntax <- pairwise.wilcox.test(jitter(results.syntax$value), results.syntax$language,
                     paired = F, p.adjust.method = "holm")
# print(p.values.syntax)
# we add some random noise here to the value vector with
# the function jitter(), since we otherwise get warnings due to ties in the data
m <- p.values.syntax$p.value
indices <- which(m < 0.05, arr.ind = T)
v1 <- rownames(m)[indices[, "row"]]
v2 <- colnames(m)[indices[, "col"]]
df <- cbind(v1, v2)
print(df)
```

```
##      v1 v2
```

Effect Size

Statistical significance is only one part of the story. For instance, a difference in complexity values might be statistically significant, but so small that it is negligible for any theorizing. In fact, it is sometimes argued that effect sizes – rather than p-values – should be the aim of statistical inquiry (Cahusac 2021, p. 12-15). An overview of effect size measures per statistical test is given in Patil (2020). In conjunction with the Wilcoxon rank sum test (there called Mann-Whitney U test) we here use the statistic r as an effect size measure (i.e. function wilcox_effsize() of the “rstatix” package). It runs from 0 to 1. Values from 0.1 to 0.3 are typically considered small effects, 0.3 to 0.5 moderate effects, and >= 0.5 large effects.

For all measures

```
effect.sizes <- wilcox_effsize(results.long, value ~ language, paired = F)
#print(effect.sizes)
```

For morphology

```
effect.sizes.morph <- wilcox_effsize(results.morph, value ~ language, paired = F)
#print(effect.sizes.morph)
```

For syntax

```
effect.sizes.syntax <- wilcox_effsize(results.syntax, value ~ language, paired = F)
#print(effect.sizes.syntax)
```

For all measures by treebank rather than language

```
# all languages
effect.sizes.treebanks <- wilcox_effsize(results.long, value ~ id, paired = F)
# selected languages with several treebanks
effect.sizes.treebanks.selected <- wilcox_effsize(results.long.selected,
                                                   value ~ id, paired = F)
```

Effect Size Heatmaps

Plot heatmaps with effect sizes for pairwise tests to get a better overview.

For all measures

```
effect.sizes.plot <- ggplot(as.data.frame(effect.sizes), aes(group1, group2)) +
  geom_tile(aes(fill = effsize), color = "white") +
  scale_fill_gradient2(low = "light blue", mid = "light grey", high = "red",
                       midpoint = 0.5, limit = c(0,1)) +
  geom_text(aes(label = round(effsize, 2))) +
  labs(x = "", y = "") +
  theme_minimal() +
  theme(axis.text.x = element_text(angle = 90, hjust = 1))
effect.sizes.plot
```

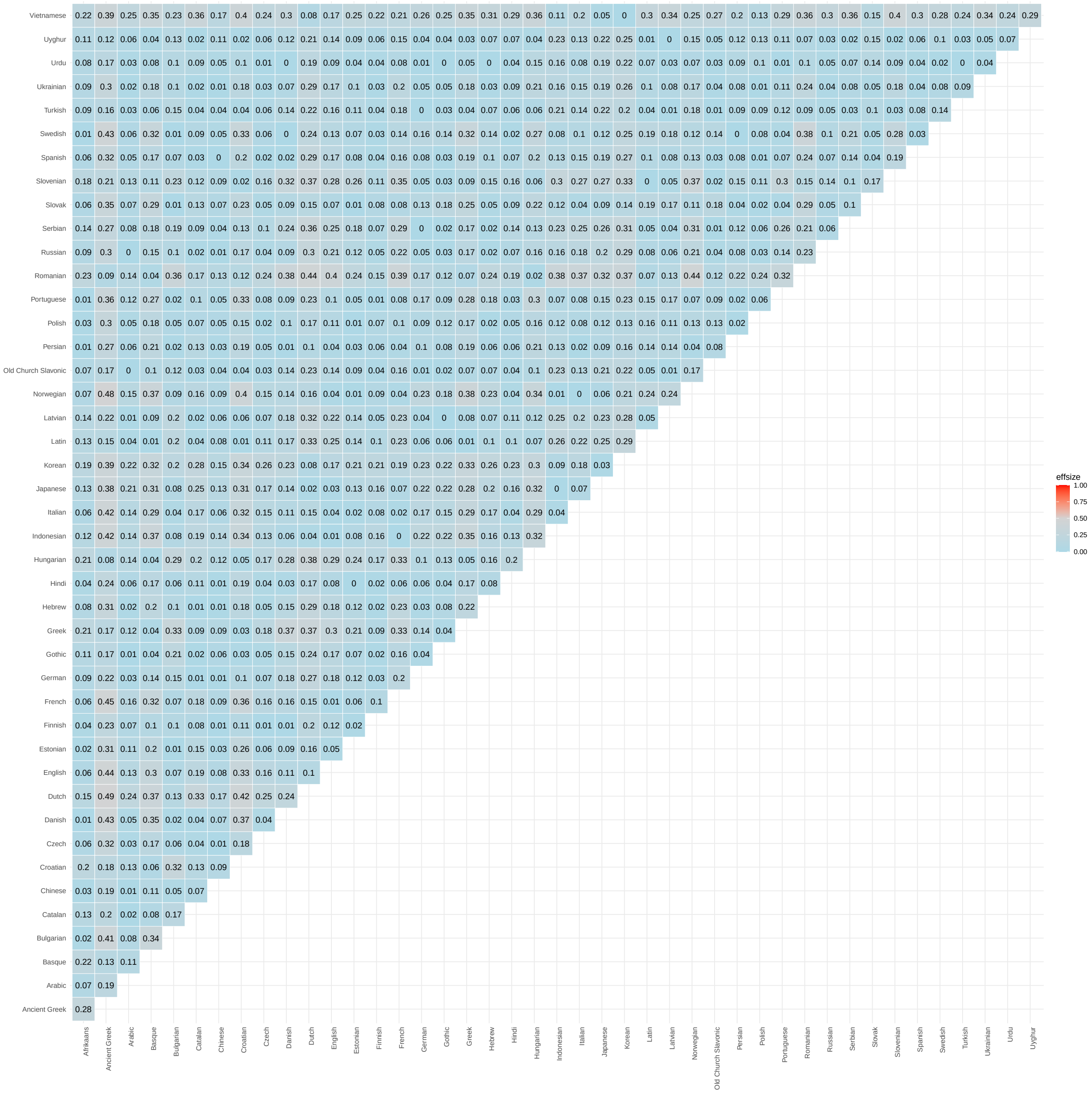

Save figure to file.

```
#ggsave("Figures/EquiCompl/effectSize_plot.pdf", effect.sizes.plot,
#       dpi = 300, scale = 1, width = 20, height = 20, device = cairo_pdf)
```

For morphology

```
effect.sizes.plot <- ggplot(as.data.frame(effect.sizes.morph), aes(group1, group2)) +
  geom_tile(aes(fill = effsize), color = "white") +
  scale_fill_gradient2(low = "light blue", mid = "light grey", high = "red",
                       midpoint = 0.5, limit = c(0,1)) +
  geom_text(aes(label = round(effsize, 2))) +
  labs(x = "", y = "") +
```

```
  theme_minimal() +
  theme(axis.text.x = element_text(angle = 90, hjust = 1))
effect.sizes.plot
```

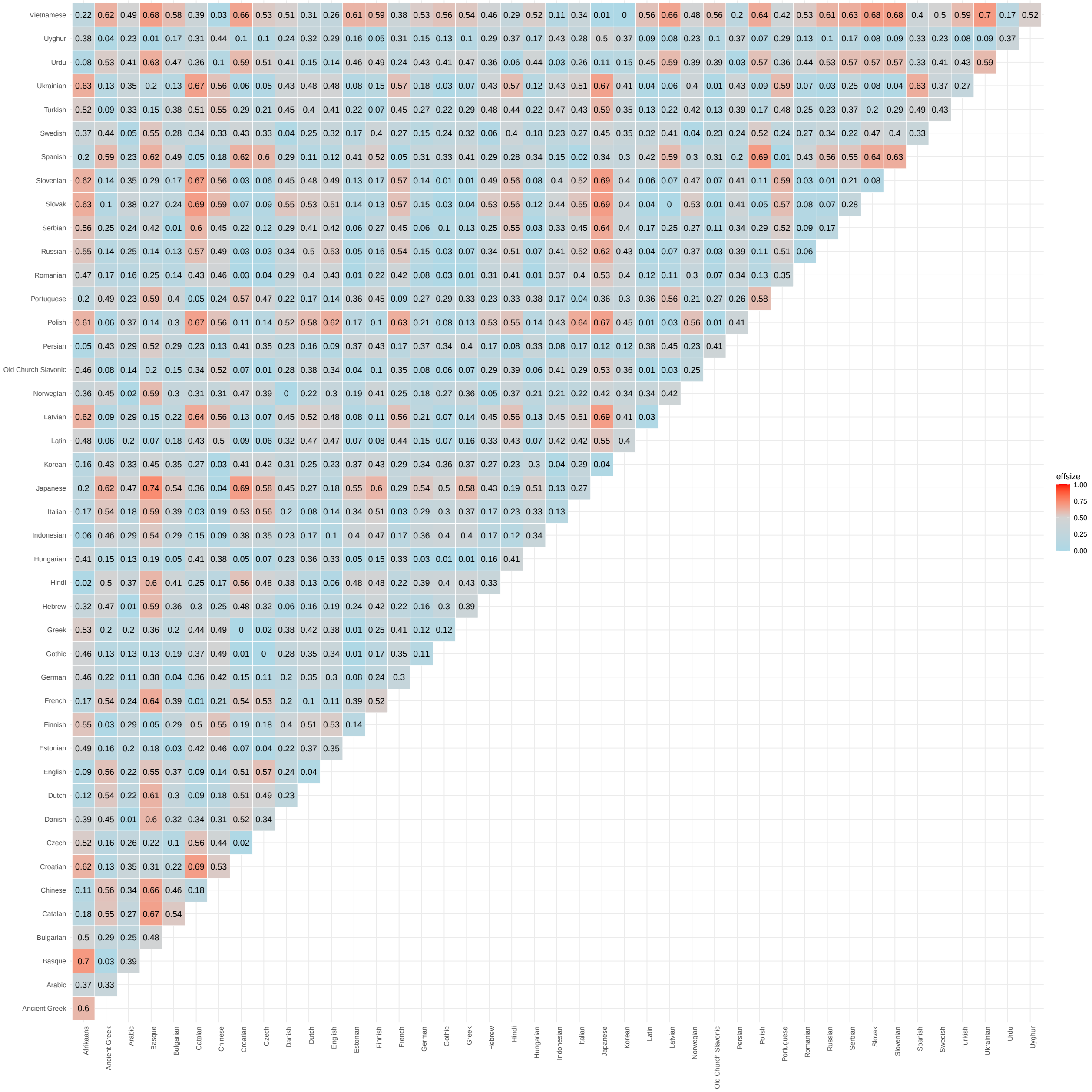

For syntax

```
effect.sizes.plot <- ggplot(as.data.frame(effect.sizes.syntax), aes(group1, group2)) +
  geom_tile(aes(fill = effsize), color = "white") +
  scale_fill_gradient2(low = "light blue", mid = "light grey", high = "red",
                       midpoint = 0.5, limit = c(0,1)) +
  geom_text(aes(label = round(effsize, 2))) +
  labs(x = "", y = "") +
```

```
  theme_minimal() +
  theme(axis.text.x = element_text(angle = 90, hjust = 1))
effect.sizes.plot
```

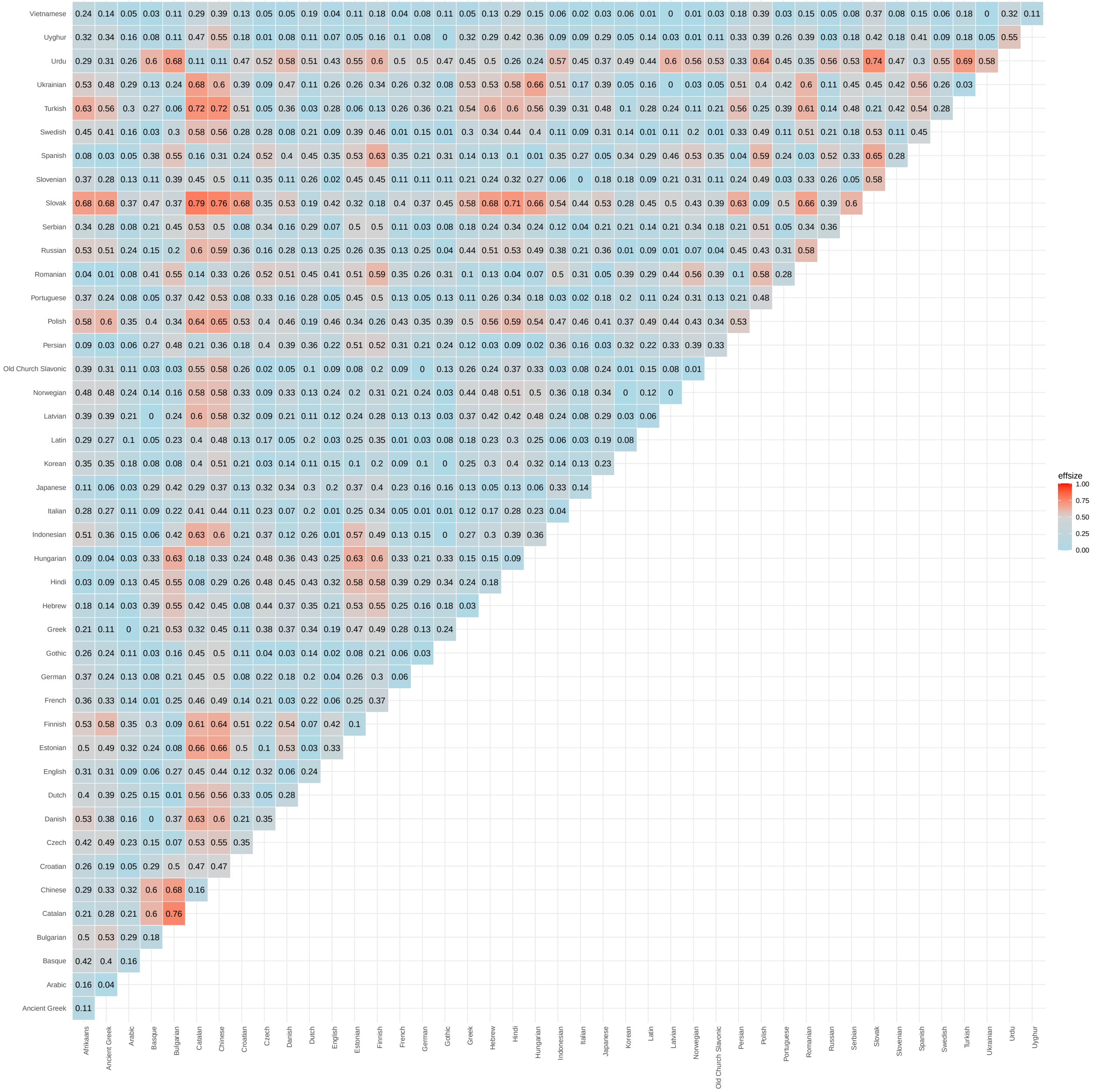

For all measures by treebank

All of the statistical analyses above are based on pairwise comparisons between languages. However, we can also look at pairwise comparisons by treebanks, since some languages are represented by several treebanks.

```
effect.sizes.plot <- ggplot(as.data.frame(effect.sizes.treebanks), aes(group1, group2)) +
  geom_tile(aes(fill = effsize), color = "white") +
  scale_fill_gradient2(low = "light blue", mid = "light grey", high = "red",
                       midpoint = 0.5, limit = c(0,1)) +
```

```r
  geom_text(aes(label = round(effsize, 2))) +
  labs(x = "", y = "") +
  theme_minimal() +
  theme(axis.text.x = element_text(angle = 90, hjust = 1))
effect.sizes.plot
```

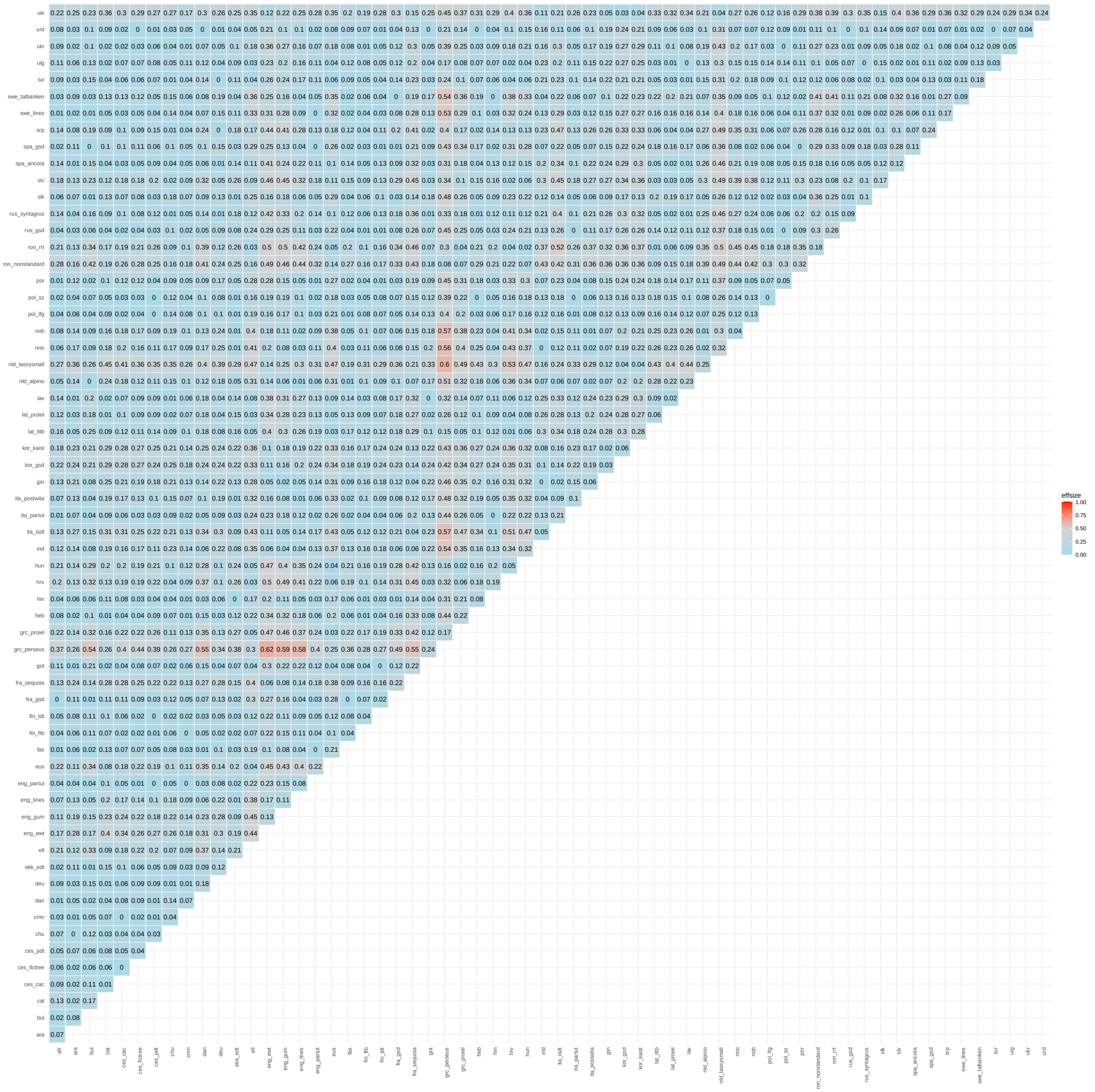

Plot heatmap for selected languages/treebanks.

```r
effect.sizes.plot <- ggplot(as.data.frame(effect.sizes.treebanks.selected),
                            aes(group1, group2)) +
  geom_tile(aes(fill = effsize), color = "white") +
  scale_fill_gradient2(low = "light blue", mid = "light grey", high = "red",
                       midpoint = 0.5, limit = c(0,1)) +
  geom_text(aes(label = round(effsize, 2))) +
```

```
  labs(x = "", y = "") +
  theme_minimal() +
  theme(axis.text.x = element_text(angle = 90, hjust = 1))
effect.sizes.plot
```

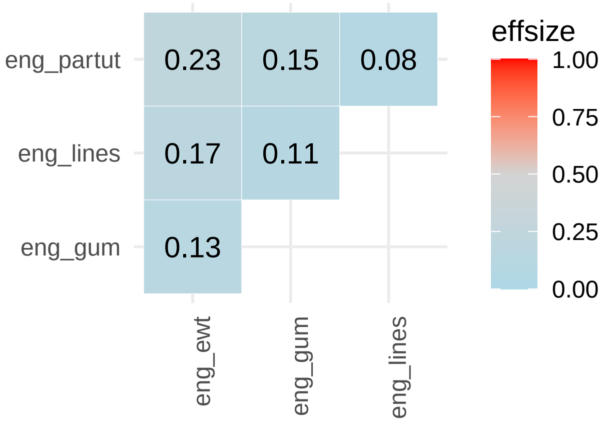

Interpretation:

We find very little evidence for systematic differences (i.e. statistically significant differences with moderate to high effect sizes) in the aggregate complexities of the 44 languages investigated here. In fact, there are only five pairs of languages out of 946 for which we find statistically significant location shifts in the overall complexity distributions. These mostly involve Ancient Greek compared to modern day languages (English, Dutch, Norwegian, and Italian). A further significant difference we find between English and Romanian.

While these results certainly depend on the number of data points (i.e. measurements) available per language, it is clearly visible in the effect size matrices that for morphology and syntax separately we find sometimes considerable differences, while for both domains aggregated there are generally low effect sizes.

The heatmap of effect sizes for English treebanks illustrates that within the same language there seems little evidence for complexity shifts depending on different styles/registers of treebanks (at least based on the UD data used here).

So, given the data, methods, and languages in this meta study, in the vast majority of cases we cannot reject the null hypothesis that languages are equally complex.

References

Cahusac, P. M. B. (2021). Evidence-based statistics. John Wiley & Sons.

McDonald, J.H. (2014). Handbook of Biological Statistics (3rd ed.). Sparky House Publishing, Baltimore, Maryland. online at http://www.biostathandbook.com

Patil, I. (2020). Test and effect size details. online at https://cran.r-project.org/web/packages/statsExpressions/vignettes/stats_details.html.
